# Cilia in the brain display region-dependent oscillations of length and orientation

**DOI:** 10.1371/journal.pbio.3003197

**Published:** 2025-07-11

**Authors:** Roudabeh Vakil Monfared, Sherif Abdelkarim, Pieter Derdeyn, Kiki Chen, Hanting Wu, Kenneth Leong, Tiffany Chang, Justine Lee, Sara Versales, Surya M. Nauli, Kevin Beier, Pierre Baldi, Amal Alachkar

**Affiliations:** 1 Department of Pharmaceutical Sciences, School of Pharmacy and Pharmaceutical Sciences, University of California-Irvine, Irvine, California, United States of America; 2 Department of Computer Science, School of Information and Computer Sciences, University of California-Irvine, Irvine, California, United States of America; 3 Mathematical, Computational, and Systems Biology Program, University of California, Irvine, California, United States of America; 4 Department of Biomedical and Pharmaceutical Sciences, School of Pharmacy, Chapman University, Health Science Campus, Chapman University, Irvine, California, United States of America; 5 Department of Physiology and Biophysics, School of Medicine, University of California, Irvine, California, United States of America; 6 Department of Neurobiology and Behavior, University of California, Irvine, California, United States of America; 7 Department of Biomedical Engineering, University of California, Irvine, California, United States of America; 8 Center for the Neurobiology of Learning and Memory, University of California, Irvine, Irvine, California, United States of America; 9 Institute for Genomics and Bioinformatics, School of Information and Computer Sciences, University of California-Irvine, Irvine, California, United States of America; Institut Curie, FRANCE

## Abstract

In this study, we conducted high-throughput spatiotemporal analysis of primary cilia length and orientation across 22 mouse brain regions. We developed automated image analysis algorithms, which enabled us to examine over 10 million individual cilia, generating the largest spatiotemporal atlas of cilia. We found that cilia length and orientation display substantial variations across different brain regions and exhibit fluctuations over a 24-h period, with region-specific peaks during light-dark phases. Our analysis revealed unique orientation patterns of cilia, suggesting that cilia orientation within the brain is not random but follows specific patterns. Using BioCycle, we identified rhythmic fluctuations in cilia length across five brain regions: the nucleus accumbens core, somatosensory cortex, and the dorsomedial, ventromedial, and arcuate hypothalamic nuclei. Our findings present novel insights into the brain cilia dynamics, and highlight the need for further investigation into cilia’s role in the brain’s response to environmental changes and regulation of oscillatory physiological processes.

## Introduction

Cilia are evolutionarily conserved, microtubule-based organelles that extend from the surface of eukaryotic cells, including neurons in the mammalian brain. Their presence in diverse organisms from single-celled organisms to complex multicellular animals emphasizes their fundamental importance in a wide range of biological processes. Primary cilia, in particular, serve as essential sensory and signaling hubs, playing critical roles in cellular homeostasis, development, and signal transduction [1–9].

A striking feature of cilia is their dynamic nature, which is manifested in the continuous modulation of their structure and function in response to various environmental cues and cellular signaling events [5,6,10–16] Mechanical forces or cerebrospinal fluid (CSF) flow can induce elongation or shortening of cilia, while intracellular signaling pathways mediated by cyclic AMP (cAMP) or calcium can reversibly modulate their morphology [17–20]. We and others have shown that neurotransmitters such as dopamine, serotonin, melanin-concentrating hormone (MCH), and adenosine can modulate primary cilia length, affecting their signaling capacity [21–24]. Although primary cilia are non-motile, their orientation can be dynamic due to the underlying cellular structures and signaling pathways regulating their relative positions. This dynamic orientation allows primary cilia to adapt to environmental changes and respond to different extracellular cues more effectively. Cilia orientation may adjust in response to numerous and varied cellular events, such as during cell migration [25,26], tissue repair [25], or in response to extracellular stimuli [27] such as mechanical stress or chemical gradients.

Many environmental stimuli and cellular signaling events sensed by cilia often follow rhythmic temporal patterns, such as light-dark cycles and fluctuations in temperature or nutrient availability [28–34]. These patterns also extend to a broad range of functions influenced by cilia signaling, such as daily sleep-wake cycles, feeding behaviors, sexual/reproductive behaviors, energy balance, metabolism, and regulation of body temperature [35–42]. Furthermore, G-protein coupled receptors (GPCRs) located on primary cilia, including melanocortin 4 receptor (MC4R), melanin-concentrating hormone receptor 1 (MCHR1), neuropeptide Y receptors 2 and 5 (NPY2R, NPY5R), somatostatin receptor 3 (SSTR3), serotonin receptor 6 (5HT6), and dopamine receptor 1 (DRD1), DRD2, and galanin receptorare involved in various rhythmic functions such as food intake, body temperature regulation, and sleep [37,43–47]. We have previously uncovered a high degree of circadian rhythmicity and spatiotemporal oscillations in cilia-associated gene expression across mouse and primate brain areas, together with dynamic shifts in the expression of genes associated with primary cilia throughout the human life span [48,49]. This was further supported by our research into cilia gene dysregulation in major psychiatric disorders, hinting at shared pathophysiological processes [50]. This implies that cilia-signaling dysfunctions may underlie common neurological deficits across these disorders.

Despite significant advancements in our understanding of primary cilia structural dynamics, evidence linking cilia dynamics to biological rhythms remains limited. This study aims to characterize the dynamic nature of primary cilia in the mouse brain by analyzing spatiotemporal variations in cilia length and orientation and assessing their time-of-day-dependent fluctuations. Through a comprehensive analysis of cilia properties and their temporal changes, we hope to provide a foundation for future studies to investigate the functional roles of cilia across brain regions and their potential contributions to time-dependent physiological processes.

## Methods

### Animals

All experimental procedures were approved by the Institutional Animal Care and Use Committee (IACUC) of the University of California, Irvine (Protocol #AUP-23-017), and were conducted in accordance with national and institutional guidelines for the care and use of laboratory animals. Eight-week-old Swiss Webster male mice were obtained from Charles River (North Carolina, USA). The mice were maintained on a 12-h light/dark cycle and provided with food and water ad libitum.

### Brain tissue collection

Mice were deeply anesthetized using isoflurane and subsequently transcardially perfused with a combination of 0.9% saline and 4% paraformaldehyde (PFA) [22]. We used a total of 56 mice, perfusing 4–5 mice at every 2-h interval over a 24-h period, resulting in 12 time points. This sample size was chosen to provide sufficient statistical power to detect significant cilia morphological differences, as validated for accuracy and reproducibility in our earlier publications. Following perfusion, we harvested the brains, preserved them in PFA overnight, and then transferred them to a 30% sucrose solution. The brains were then coronally sectioned at a thickness of 30 μm using a microtome.

### Immunohistochemistry and imaging

For immunohistochemistry preparation, 3−4 sections were selected from various levels across the brain. The sections were then blocked using goat serum in PBS containing 0.3% Triton X-100 for 1 h. Following this step, the brain sections were incubated in blocking buffer solution containing the rabbit primary anti-adenylate cyclase 3 (ADCY3) antibody (LSBIO-C204505), at a dilution of 1:500. Post-primary antibody incubation, the sections were rinsed with PBS and incubated with the secondary antibody, Invitrogen AlexaFluor546 goat anti-rabbit, at a dilution of 1:500, along with DAPI at a dilution of 1:10,000 (Thermo Fisher Scientific). The sections were then washed with PBS and mounted onto slides. The cilia were imaged using the Keyence BZ-9000 fluorescence microscope with a 20× objective lens. Brain regions analyzed include: Anterior hypothalamic area (AHC), Arcuate nucleus (ARC), Dorsomedial hypothalamus (DMH), Lateral hypothalamus (LH), Median preoptic area (MPO), Hypothalamic paraventricular nucleus (PVN), Suprachiasmatic nucleus (SCN), Supraoptic nucleus (SON), Ventromedial hypothalamus (VMH), Caudal striatum (CS), Nucleus accumbens core (NAc), Nucleus accumbens shell (NAs), Rostral striatum (RS), Olfactory tubercle (TU), Cornu Ammonis 1 (CA1), Cornu Ammonis 3 (CA3), Dentate Gyrus (DG), Motor cortex (MC), Prefrontal cortex (PFC), Piriform cortex (Pir), Somatosensory cortex (SSC), Medial amygdala (MA). Dual-staining immunohistochemistry was performed to verify the base and tip of primary cilia. The basal body was detected using a rabbit anti-Centrin 1 polyclonal antibody (Proteintech, 12794-1-AP) at a 1:200 dilution, while the cilia were stained with a chicken anti-ADCY3 antibody (EnCor Bio, CPCA-ACIII) at a 1:500 dilution. Secondary antibodies included Alexa Fluor 488 goat anti-rabbit (Invitrogen, A-11008) and Alexa Fluor 564 goat anti-chicken (Invitrogen, A-11040), both at a 1:200 dilution. Images were captured using fluorescence microscopy at 40x magnification.

### Cilia length and angle measurement

Our first step involved pre-processing the images by converting them to grayscale, to make them conducive for analysis and to mitigate color-related complications. We then applied Gaussian blur to the images to minimize noise or minor details that might compromise the accuracy of our measurements. Next, we used canny edge detection to find all the edges in the images. This allowed us to identify the cilia more easily and accurately. We applied a threshold value to the canny edge detection output to obtain a binary image where the edges were represented by white pixels and the background is represented by black pixels. After obtaining the binary image, we used the findContours() function in OpenCV to detect all the cilia in the image. This function identifies and segments all the distinct objects in the image, in this case, the cilia, and returns a list of their contours. However, some of these contours may be noise or not relevant to our analysis. Therefore, we filtered them based on their size, aspect ratio, and other factors to only capture the ones that are likely to be cilia. For instance, we filter the contours based on their length, as cilia are typically longer than they are wide. We set a minimum and maximum length threshold for the contours to keep only the ones that fall within this range. In our implementation, we use a minimum length of 1 µm and a maximum length of 15 µm. Furthermore, we filter the contours based on their aspect ratio, as cilia are generally slender structures with a length-to-width ratio greater than 3:2. We set a minimum aspect ratio threshold for the contours to remove any that do not meet this criterion. In our implementation, we used a minimum aspect ratio of 1.5. For each contour, we fit a bounding box around it using the cv2.boundingRect() function. This gave us the length and angle of the cilia. The length was calculated as the distance between the top and bottom points of the bounding box, and the angle was calculated as the angle between the major axis of the box and the horizontal axis. However, we also needed to determine the base of the cilia accurately. To achieve this, we used the cv2.minMaxLoc() function to find the brightest pixel in the grayscale image within the bounding box of the cilia. The cilia’s base is typically the most illuminated section, and this method enables us to pinpoint it with precision. To further improve the accuracy of our angle measurements, we combine the location of the base of the cilia with the center point of the bounding box. By comparing the relative positions of these points, we were able to determine which quartile the cilia were facing and adjust the angle measurement accordingly. Finally, we saved all the information about the cilia, including their lengths, angles, and positions, in a CSV file. This allowed us to analyze the data more easily and systematically. The two programs we developed and used are provided as supplementary materials (S1 Code), referred to as Program 1 and Program 2. A detailed, step-by-step description of the analysis pipeline is outlined below, and a visual walkthrough demonstrating each step is available in supplementary materials (S1 Video).

### Analysis pipeline

The cilia detection pipeline consists of three main phases: contours extraction, contours filtering, length, and angle measurement. The specific OpenCV functions used for each step are included.

#### Phase 1: Contours extraction.

1. Canny Edge Detection:
  - Purpose: Detect edges in the image, highlighting regions of rapid intensity change, which often correspond to object boundaries.
  - Method: The grayscale image is processed using cv2.Canny edge detection. The Canny function takes two thresholds to detect strong and weak edges. The lower threshold is calculated as 0.67 * median intensity, and the upper threshold is slightly increased to detect a broader range of edges.
2. Close holes
  - Purpose: Fill small holes in the binary image to create more complete and connected foreground regions.
  - Method: Morphological closing is applied using cv2.morphologyEx with the cv2.MORPH_CLOSE operation and a rectangular kernel of size (2, 2).
3. Erode edges
  - Purpose: Refine the detected edges by removing small noise and shrinking the boundaries of detected regions slightly.
  - Method: Erosion is applied using cv2.erode with a rectangular kernel of size (1, 1) for one iteration, which helps clean up the edges of the detected contours.
4. Find contours
  - Purpose: Identify the boundaries of the foreground regions, which represent the cilia structures.
  - Method: Contours are extracted from the processed binary image using cv2.findContours with cv2.RETR_EXTERNAL and cv2.CHAIN_APPROX_SIMPLE.

#### Phase 2: Contours filtering.

1. Minimum and maximum length:
  - Purpose: Ensure that only contours of appropriate length are considered, excluding those that are too short or too long.
  - Method: The length of each contour is calculated using the dimensions from cv2.minAreaRect, and contours with a length greater than 1 µm and less than 15 µm are retained.
2. Minimum length-to-width ratio:
  - Purpose: Filter out contours that do not meet a minimum aspect ratio, ensuring that only elongated structures, which are characteristic of cilia, are included.
  - Method: For each contour, the ratio of its length to width is calculated. Contours with a ratio greater than or equal to 1.5 are kept, while those below this threshold are discarded.

#### Phase 3: Length and angle measurement.

1. Getting length and angle for each contour:
  - Purpose: Measure the initial length and angle of each cilium to provide quantitative data on their orientation and size.
  - Method: For each filtered contour, the length and width are extracted using cv2.minAreaRect. The cv2.contourArea function calculates the area, and the cv2.minAreaRect function provides the minimum bounding rectangle, from which the dimensions and initial angle are derived.
2. Adjusting the angle based on base and tip prediction:
  - Purpose: Refine the initial angle measurement by determining the base and tip of the cilium for more accurate orientation data.
  - Method: To refine the angle measurement, each cilium is drawn separately on a mask, and the location of the maximum intensity within the mask is found using cv2.minMaxLoc. The purpose is to find the brightest spot within the cilium, which usually indicates the base. The relative position between the base to the center is used to adjust the angle appropriately, ensuring the measured angle reflects the true orientation of the cilium.

### Circular angles’ mean calculation

Due to the circular nature of angles, the traditional arithmetic mean method is not applicable for calculating angle means. Therefore, we computed the circular mean, also known as the angular mean, to calculate meaningful statistics for our angle dataset. The circular mean of our angle dataset was calculated as follows: First, the angle values were converted to radians, and all the data were subsequently transformed into sines and cosines. The means of the sines and cosines were calculated by summing the values and dividing them by the total count of each value. The circular mean was determined using the atan2 function, a variant of the arctangent function that accepts two arguments and yields results within the appropriate quadrant. The obtained results were then converted back to degrees using the equation: Degrees = radians × (180/*π*). If negative outputs were obtained from the atan2 function and the data ranged from 0 to 360, 360 was added to those values to align them within the correct range.

### Quantification of cilia density across mouse brain

To determine the cilia density in each of the 22 brain regions, we calculated the percentage of ADCY3-stained cilia relative to the number of DAPI-stained cells within the same region.

In order to detect cells in DAPI-staining images, the pipeline processes images to detect and analyze contours, focusing on the identification of significant structures within the image. The methodology consists of three main phases: contours extraction, contours clustering, and contours filtering.

#### Phase 1: Contours extraction.

1. Image equalization:
  - Purpose: Enhance the contrast of the grayscale image to improve the visibility of features.
  - Method: Apply Contrast Limited Adaptive Histogram Equalization (CLAHE) using OpenCV’s cv2.createCLAHE function with clipLimit=2.0 and tileGridSize=(8, 8). This method adjusts the image contrast in localized regions, enhancing the structural details.
2. Adaptive thresholding
  - Purpose: Convert the grayscale image into a binary image to segment the foreground from the background.
  - Method: Use OpenCV’s cv2.adaptiveThreshold function with parameters maxValue=255, adaptiveMethod=cv2.ADAPTIVE_THRESH_MEAN_C, thresholdType=cv2.THRESH_BINARY, blockSize=41, and C=-10. This technique applies a threshold based on the local mean intensity, effectively segmenting the image.
3. Distance transform
  - Purpose: Compute the distance to the nearest zero pixel for each pixel in the binary image to prepare for isolating the foreground objects.
  - Method: Implement OpenCV’s cv2.distanceTransform function with distanceType=cv2.DIST_L2 and maskSize=3. This transformation aids in distinguishing foreground components.
4. Thresholding:
  - Purpose: Refine the binary image to emphasize significant foreground regions.
  - Method: Apply OpenCV’s cv2.threshold function with thresh=0.2, maxval=1.0, and type=cv2.THRESH_BINARY. This step applies a fixed threshold to the distance-transformed image to highlight the foreground areas.
5. Dilation
  - Purpose: Expand the foreground regions to ensure connectivity of close components.
  - Method: Use OpenCV’s cv2.dilate function with a kernel of np.ones((3, 3), np.uint8) and iterations=1. This morphological operation enlarges the foreground regions, making them more pronounced and connected.
6. Isolate foreground and background:
  - Purpose: Separate the sure foreground, sure background, and unknown regions for better segmentation.
  - Method: Identify the sure foreground by dilating the thresholded image, and the sure background by inverting and dilating the foreground mask using cv2.bitwise_not and cv2.dilate with kernel=np.ones((3, 3), np.uint8) and iterations=1. The unknown region is derived by subtracting the sure foreground from the sure background using cv2.subtract.
7. Extract contours from foreground:
  - Purpose: Identify the boundaries of the foreground objects.
  - Method: Extract contours from the sure foreground mask using OpenCV’s cv2.findContours function with mode=cv2.RETR_EXTERNAL and method=cv2.CHAIN_APPROX_SIMPLE. This step identifies the boundaries of the foreground objects for further analysis.

#### Phase 2: Contours clustering.

1. Cluster contours with DBScan:
  - Purpose: Group closely situated contours to manage fragmented contours representing a single object.
  - Method: Use the DBSCAN (Density-Based Spatial Clustering of Applications with Noise) algorithm from scikit-learn with parameters eps=30 and min_samples=1 to cluster the contours based on their centroid positions. This clustering helps in grouping related contours.
2. Merge contours within each cluster:
  - Purpose: Combine contours within each cluster to form a single, cohesive contour for each detected object.
  - Method: Merge contours by calculating the convex hull of all points within each cluster using OpenCV’s cv2.convexHull. This step ensures that all points in a cluster are enclosed in a single contour.

#### Phase 3: Contours filtering.

1. Minimum and maximum area:
  - Purpose: Ensure that only contours of relevant size are considered for analysis.
  - Method: Filter contours based on area (in pixels) using OpenCV’s cv2.contourArea function with minimum area = 20 pixels and maximum area = 1000 pixels. This step excludes contours that are too small or too large to be relevant.
2. Minimum circularity:
  - Purpose: Eliminate contours that do not meet the minimum shape criteria, focusing on more circular objects.

Method: Calculate the circularity of each contour and retain only those with a circularity above the specified threshold using OpenCV’s cv2.arcLength function and a minimum circularity of 0.01. Circularity is calculated as:


$$circularity=\frac{4\pi \cdot Area}{{Perimeter}^{2}}$$

### Verification of the automated measurements of cilia lengths and angles

To validate the automated measurements of cilia lengths and angles, and to verify the accuracy of our pipeline in identifying and measuring cilia structures, we conducted manual checks on random samples, selecting at least 10 cilia from each of the 22 brain regions. The measurements were performed blindly using both manual and automated methods. We then compared the results by calculating the recall and mean absolute error (MAE) for both length and angle measurements.

Since cilia length and orientation were measured from 2D images, projecting 3D structures like cilia onto a 2D plane can introduce measurement errors. To address this, we estimated the potential errors introduced by this approach using basic geometric principles, including the Pythagorean theorem. Detailed calculations are provided in the supplementary material (Figs A–C in S1 Text).

### Comparative and correlational analysis of cilia lengths and angles

We analyzed the data using two different approaches: first, by assessing the lengths and angles of individual cilia to provide a detailed frequency distribution across all examined brain regions; and second, by calculating the mean length and angle of cilia in each section of the left and right hemispheres, treating these averages as single values for further analysis.

To analyze differences in cilia lengths and angles across various brain regions at different times of the day, we performed one-way ANOVA. Multiple comparisons were adjusted by controlling the False Discovery Rate (FDR) using the two-stage linear step-up procedure of Benjamini, Krieger, and Yekutieli. Additionally, we computed the Pearson correlation coefficient (*r*) for each pair of region-specific cilia lengths and angles across 24-h time points, generating a correlation matrix to examine the relationships among all brain regions. Data were analyzed using GraphPad Prism 9.4, with statistical significance set at *p* < 0.05, and expressed as mean ± standard error of the mean (SEM).

### Network analysis

Using the Allen Mouse Brain Connectivity Atlas data [51], we built a structural network of the regions used in our study. We normalized projections from source regions to other regions of interest by dividing the projection density by the injection volume. The normalized projection density was averaged across all experiments for each pair of source and target regions to generate a connectivity matrix. These values were log normalized to produce a more effective distribution for use as edge weights in a network. Community detection was carried out using the Louvain algorithm implemented in scikit-network with a resolution parameter of 1 [52]. We identified three main clusters of brain regions: purple (NAc, NAs, and others), green (PFC, MC, and others), and red (CA1, CA3, and DG). We examined the correlation between cilia length and angle with various network features. We examined all pairs of regions within and across different clusters, identifying varying fractions of region pairs correlated by either cilia length or angle. We also analyzed whether these fractions would likely occur if regions were correlated randomly, shuffling all correlations across region pairs for this purpose. We constructed cilia correlation networks using a log-scaled *R* value to achieve a desirable distribution for edge weights. Network analysis was performed in Python using NetworkX [53], with network visualizations created using Plotly [54].

### Circadian rhythmicity analysis

BIO_CYCLE, a deep-learning model designed for assessing periodicity of signals, was utilized to determine significant circadian patterns in cilia length and angle, as well as their oscillation’s amplitude and phase [55,56]. BIO_CYCLE is trained using synthetic and real biological time series datasets with labeled periodic and aperiodic signals. This tool comprises a deep neural network (DNN) for classification, which distinguishes periodic from non-periodic signals, and another DNN for regression estimating the signal’s period, phase, and amplitude. The oscillation status of a parameter is defined based on a *p*-value (with a cutoff of 0.05) computed by BIO_CYCLE as follows: *N* aperiodic signals are first generated from the synthetic time series datasets, and the *N* output values *V*(*i*) (*i* = 1, …, *N*) of the classification DNN on these aperiodic signals are calculated. The *N* output values of the classification DNN on these aperiodic signals are then calculated, forming the basis for the null hypothesis distribution. The output value *V* of the new signal s is then compared to *V*(*i*) (*i* = 1, …, *N*), producing the estimate for the probability of obtaining an output of size *V* or greater (*p*-value), assuming that the signal is part of the null distribution (aperiodic signals). A smaller *p*-value suggests a higher likelihood of the signal being periodic. The related *q*-values are calculated via the Benjamini and Hochberg procedure. BIO_CYCLE is freely accessible from the CircadiOmics web portal: http://circadiomics.igb.uci.edu.

## Results

**Fig 1:**
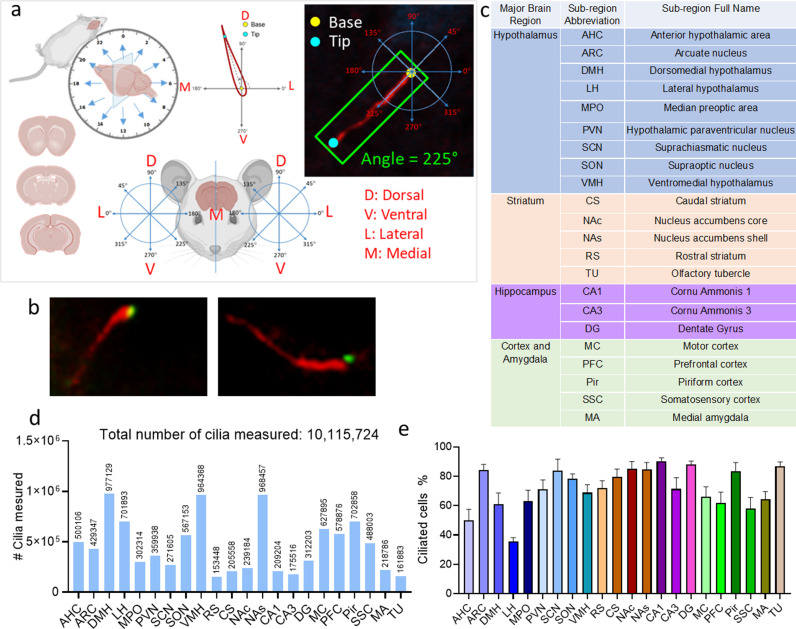
Experimental design and data analysis. **(A)** Schematic Representation: (Top Left) Mice were perfused and harvested at two-hour intervals over a 24-h period. The harvested mice were then sectioned coronally. (Bottom Left) Coronal brain sections at different brain levels. (Top Middle) The ciliary base was defined as the origin of the coordinate system, and the tip of the cilia was labeled. Cilia length was measured from the base to the tips, and the circular angle was determined. (Bottom Middle) The brain section of the mice was divided into left and right hemispheres. For cilia found in the right hemisphere, a counter-clockwise coordinate system was used to determine the circular angle (Right). For cilia found in the left hemisphere, a clockwise coordinate system was used (Left). (Top Right) An example of the angle measurement from cilia found in the right hemisphere. **(B)** Representative images of immunofluorescence staining showing cilia labeled with the ADCY3 antibody (red) and the basal body (cilia base) labeled with the centrin 1 antibody (green). **(C)** Brain Regions analyzed for cilia length and angle. **(D)** The number of cilia analyzed in 22 different brain regions of the mice; underlying data are available on the Zenodo database (https://doi.org/10.5281/zenodo.15151271). **(E)** Cilia density in 22 brain regions, expressed as the percentage of ciliated cells relative to the total number of cells in each region, underlying data are available in S1 Data. Parts of Fig 1A are created with BioRender.com.

We mapped the spatiotemporal patterns of cilia length and orientation across various brain regions in mice. We developed and used a special program to analyze cilia in brain sections from a large cohort of over 50 mice, with a minimum of four mice at each of the twelve 2-h intervals within a 24-h cycle (Fig 1A and Fig D in S1 Text). We verified the cilia base through dual immunostaining of Centrin 1, a well-established marker for the ciliary basal body, and ADCY3 (Fig 1B). We examined 3–4 brain sections per region at each time interval across 22 cilia-rich brain regions (Fig 1C). Our automated method allowed us to analyze 10 million cilia and more than 20 million data points, with counts of cilia assessed in specific regions ranging from 153,448 in the RS to 977,129 in the DMH (Fig 1D). The original raw data for these analyses are available in the Zenodo database (https://doi.org/10.5281/zenodo.15151271).

### Methodological verification

To validate the accuracy of our automated system for measuring cilia length and angle, we conducted a comparative analysis with manual measurements. In each of the 22 brain regions analyzed, 10 cilia were measured manually. The average of these manual measurements was then compared to the automated measurements. This comparative analysis showed a recall of 93.46%, indicating high reliability in the automated detection of cilia. The MAE for cilia length was 1.33 µm, and the MAE for angle measurements was 7.759 degrees (S1 Data). We also estimated the instrumental error introduced by measuring 3D cilia structures in 2D Detailed calculations are provided in the Supplementary results (Figs A–C in S1 Text).

### Region-dependent variations in cilia density, length, and angle in the mouse brain

Our analysis demonstrated marked variability in cilia density across the 22 brain regions examined. The lowest density was observed in the LH, where only 35% of cells were ciliated, while the highest density was found in the CA1 region of the hippocampus, with 90% of cells containing cilia (Fig 1E and S1 Data).

We assessed the average cilia length within specific brain regions using two distinct approaches. First, in the direct regional average method, we calculated the average length of each cilium individually within each region, revealing a variation in cilia lengths from 4.79 µm in the NAs to 6.33 µm in the TU (panel a of Fig E in S1 Text). Across all brain regions, including over 10 million cilia, this method resulted in an overall mean cilia length of 5.15 µm.

**Fig 2:**
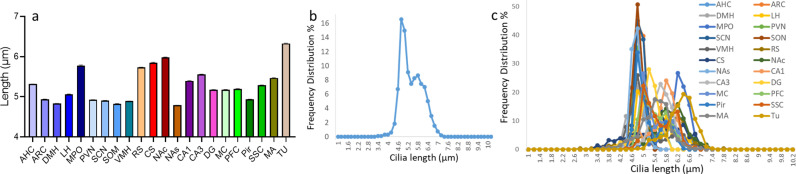
Cilia length analysis in different brain regions. **(A)** Histogram showing the means±SE of cilia length in the 22 brain regions based on the average length measured in each brain section. The x-axis represents cilia length in micrometers and the y-axis represents the percentage frequency of occurrence. **(B** and **C)** Histograms showing the frequency distribution of cilia length in **(B)** the entire brain and **(C)** the 22 individual brain regions, using the section-based average method. For each brain section, the lengths of all cilia were averaged to obtain a single value, which was then used to calculate the overall mean cilia length for each region. The mean cilia length was derived from the average lengths of both left and right hemisphere sections (3–4 sections per region). The x-axis represents cilia length in micrometers, and the y-axis shows the percentage frequency of occurrence; underlying data for Fig 2 are available in S2 Data.

Second, in the section-based average method, we calculated the average of lengths of all cilia within each section to obtain a single value per section, then used these sections’ averages to calculate the overall mean cilia length for each region. The brain-wide cilia length average was 5.4 ± 0.01 µm, with regional averages ranging from 4.87 µm in the NAs to 6.3 µm in the TU (Fig 2A and S2 Data). Additionally, a frequency distribution analysis showed that approximately 40% of cilia across all examined regions had lengths within the 4.8–5.4 µm range (Fig 2B and 2C). However, the frequency distribution analysis revealed a varied distribution of cilia lengths across the 22 brain regions. Some regions, like the ARC, MPO, SCN, and NAs have most cilia lengths concentrated around a narrow range, whereas other regions, like the CS and RS, exhibit a broader distribution, with cilia lengths spread across a wider range (panel b of Fig E in S1 Text).

**Fig 3:**
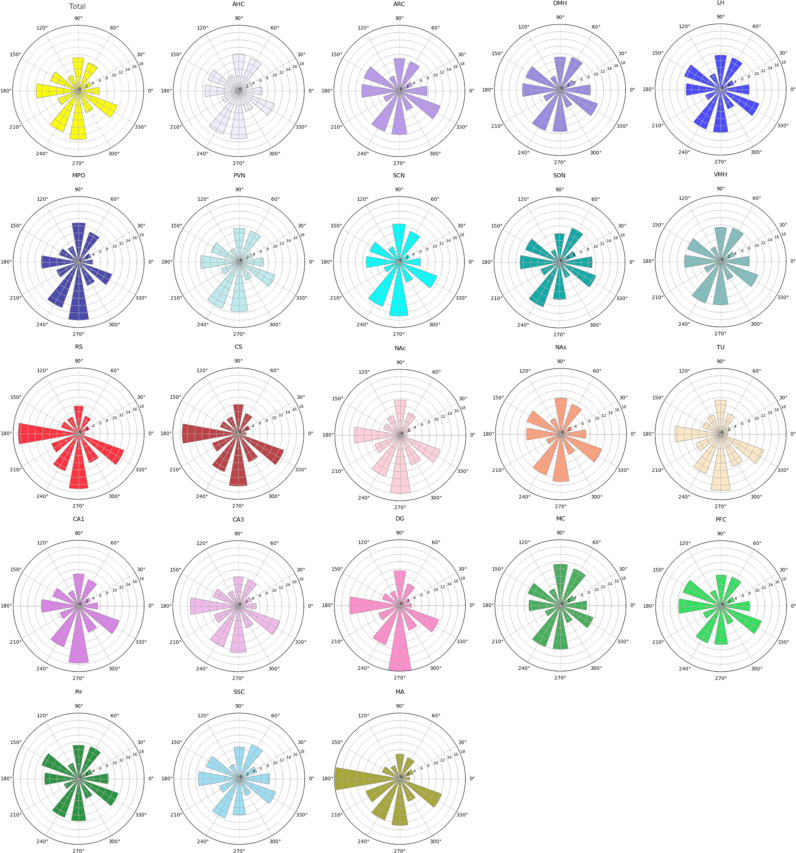
Cilia circular angle analysis, based on individual cilia measurements. Rose plot displaying the frequency distribution of cilia circular angles in individual brain regions from all individual measured cilia at 30° bins, Underlying data are available on the Zenodo database (https://doi.org/10.5281/zenodo.15151271).

Our analysis of individual cilia angles of over 10 million cilia showed a distinct pattern across all regions (Fig 3 and Fig F in S1 Text). Notably, the highest distribution of cilia orientation across the entire brain was toward 180° and 270°, indicating a primary medial and/or ventral orientation of cilia (Fig 3 and Fig F in S1 Text). However, cilia orientation did not exhibit uniform patterns across different brain regions (Fig 3). The RS, CS, MA, and CA3 showed a predominant cilia orientation toward 180°, and a secondary preference for 270°, whereas the NAc, TU, MPO, SCN, DG, and CA1 exhibited a primary orientation toward 270°, followed closely by 180° (Fig 3). A more scattered distribution of cilia orientation was observed in the hypothalamic nuclei, various cortices, and the NAs.

**Fig 4:**
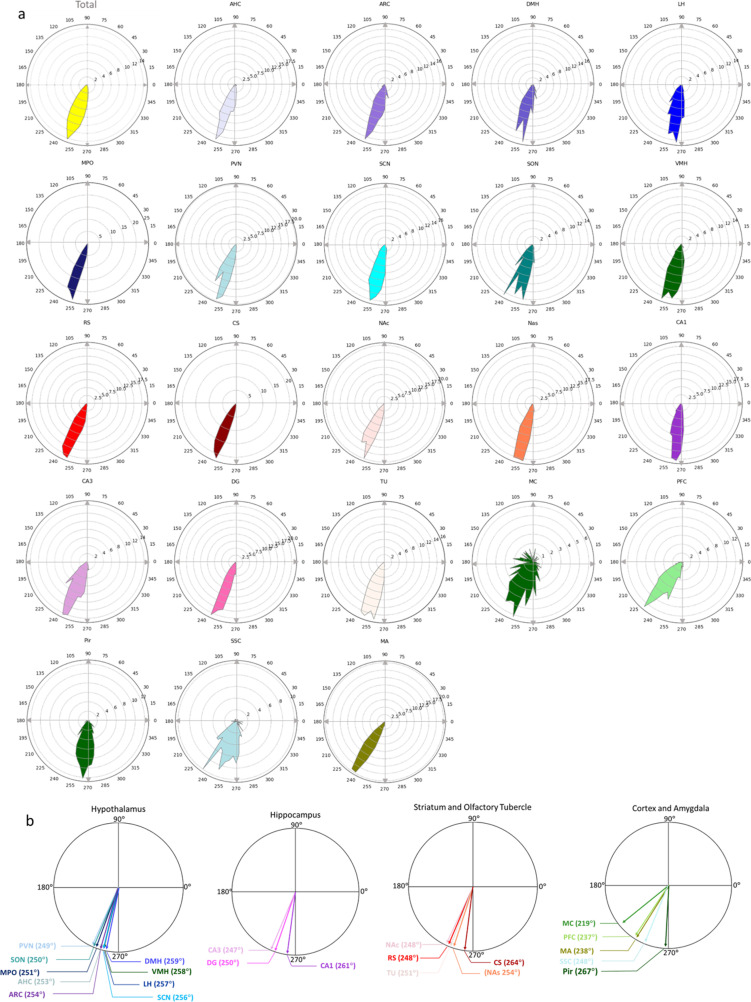
Cilia circular angle analysis based on section-averaged circular means. **(A)** Radial plot showing the frequency distribution of cilia circular angles across the entire brain and individual brain regions. The analysis is based on section-averaged circular means, where the circular mean of all cilia angles within each section was first calculated and treated as a single value. Underlying data are available in S2 Data. **(B)** Radial plots displaying the mean cilia circular angles for the 22 brain regions, grouped into major categories: hypothalamus, hippocampus, cortices, and striatum. The average angle for each region was determined by calculating the circular angle means for each brain section. Underlying data are available in S2 Data.

When using the section-based averaging, where the circular mean of all cilia angles within each section was first determined and treated as a single value, cilia angle means and distributions revealed distinct patterns. Cilia angles predominantly favored the third quadrant (180°–270°), particularly around 250°, when measured relative to the horizontal line in coronal sections (Fig 4A, 4B and panel a of Fig G in S1 Text). The Pir exhibited the highest circular mean cilia angle (267°), whereas the MC displayed the lowest (219°) (Fig 4B and panel b of Fig G in S1 Text).

We found no significant correlation between cilia density and cilia length (Pearson’s coefficient, *r* = −0.08, *P* = 0.718), whereas there was a trend toward a positive correlation between cilia density and orientation (*r* = 0.38, *P* = 0.084).

### Temporal fluctuations and rhythmicity in cilia length and orientation across brain regions

**Fig 5:**
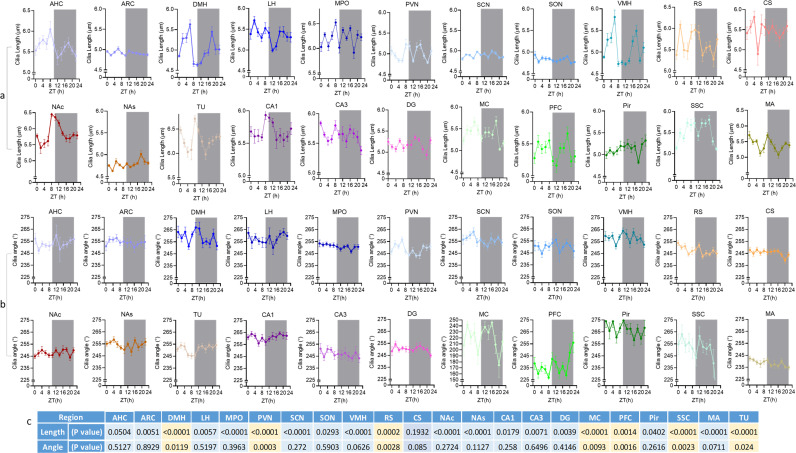
Fluctuation of cilia length and cilia circular angle across 24-hours’ time. **(A and B)** Diurnal fluctuations of cilia length **(A)** and angle **(B)**: The plots show the means ± SE of cilia length and angle over the course of 24-h day in different brain regions. The brain regions are grouped into major categories including, the Hypothalamus, Hippocampus, Cortices, and Striatum. The average length and angle are determined based on the measurements’ means in each brain section; Zeitgeber time (ZT). One-way ANOVA test was used to compare the means of lengths and angles at different time points. *P* values were calculated using the False Discovery Rate (FDR) correction, employing the Two-stage linear step-up procedure of Benjamini, Krieger, and Yekutieli for multiple comparisons. **(C)** P values of one-way ANOVA test, used to assess the variability in cilia length and angle at different time points (in a,b), *P* < 0.05: significant changes across the different time points over 24-h period. Underlying data for Fig 5 are available in S2 Data.

We next analyzed cilia lengths and orientations at 2-h intervals over a 24-h period (S2 Data), and found complex, time-dependent fluctuations in both cilia length and orientation across various brain regions (Fig 5A and 5B). Notably, all regions except the AHC and CS exhibited significant variations in cilia length, with distinct fluctuation patterns observed during the light and dark phases (Fig 5A–5C).

Notably, the MA, LH, RS, CS, TU, AHC, CA3, and NAc reached their maximum cilia lengths at specific zeitgeber times (ZTs) during the light phase, whereas the ARC, DG, NAs, and PFC showed peak lengths at different ZTs during the dark phase (Fig 5A and 5C). Interestingly, both the DMH and VMH exhibited a bimodal pattern, with cilia length peaks occurring at ZT 6 and ZT 18 during the light and dark phases, respectively (Fig 5A and 5C).

We observed significant time-dependent fluctuations in cilia angles within seven brain regions: DMH, PVN, RS, MC, PFC, SSC, and TU (Fig 5B and 5C). Notably, the DMH and PVN, two hypothalamic nuclei, displayed contrasting cilia angle patterns. The DMH reached its highest cilia angle between ZT 16–20, while the PVN showed its lowest angle during this same period. Conversely, at ZT 6, the DMH exhibited its lowest angle, and the PVN reached its highest (Fig 5B and 5C). The PFC’s cilia angle changes were opposite to those in the SSC, with the PFC peaking at ZT 22, while the SSC reached its lowest angle at that time. The MC showed the most extensive range of cilia angle shifts, varying from 174° at ZT 20°–245° at ZT 16 (Fig 5B and 5C).

**Fig 6:**
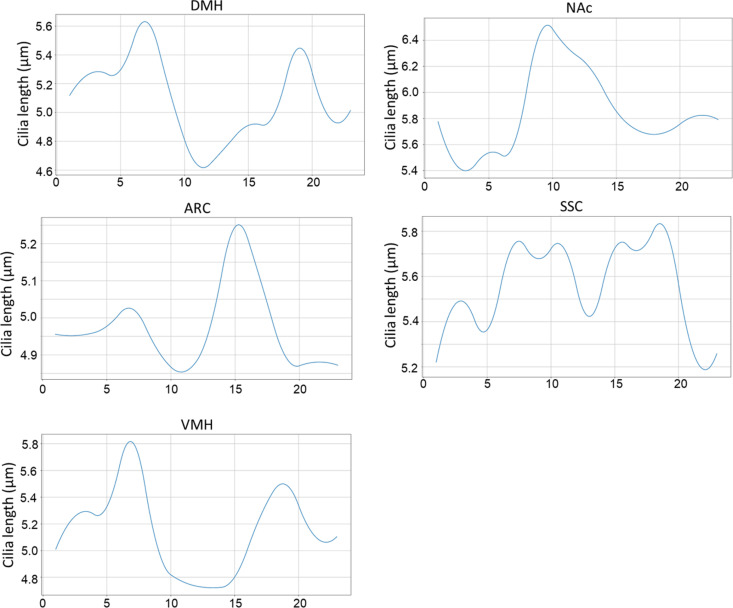
Circadian rhythms of cilia length. BioCycle analysis was used to examine the presence of rhythmic fluctuations in cilia length and revealed significant circadian patterns of cilia length variations across a 24-h period in five brain regions tested: ARC, DMH, VMH, NAc, and SSC, with *P* < 0.05. Underlying data are available in S3 Data.

Our analysis revealed significant periodic fluctuations in cilia length within five specific regions: ARC, DMH, VMH, NAc, and SSC, with peak times varying among them: ARC at ZT 15, DMH at ZT 3.8, VMH at ZT 3.4, NAc at ZT 11, and SSC at ZT 12.16 (Fig 6). However, we found no evidence of rhythmic patterns in cilia angles in any of the regions studied (S3 Data).

### Correlation of cilia length and circular angle among various brain regions

**Fig 7:**
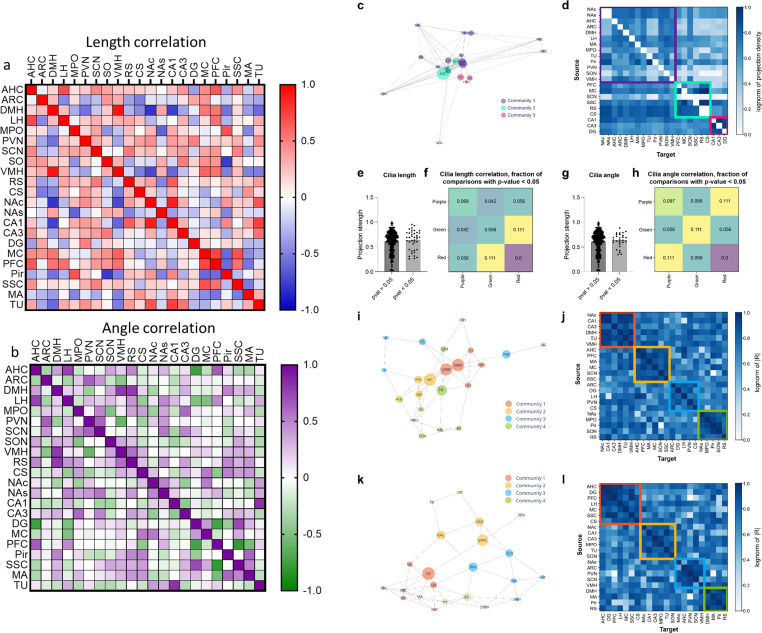
Correlation of cilia length and circular angle among various brain regions and relationship between cilia length/angle and network connectivity. **(A)** Heatmap showing the correlation of cilia length (based on the mean of the section means) among different brain regions. The correlation coefficient ranges from 1 (indicating the most positive correlation, red) to −1 (indicating the most negative correlation, blue). Positive correlation implies that the cilia lengths in the two regions are on the longer and shorter side simultaneously. Negative correlation indicates that the two regions have opposite cilia length sizes; underlying data are available in S2 Data. **(B)** Heatmap displaying the correlation of cilia angle (based on the mean of the section circular means) among different brain regions. The correlation coefficient ranges from 1 (indicating the most positive correlation, purple) to −1 (indicating the most negative correlation, green); underlying data are available in S2 Data. **(C)** Anatomical network of the brain regions in our study. The network is embedded in 2D space using a spring embedding, in which highly connected nodes are pulled together as by a spring. Node size represents betweenness, a measurement of centrality based on the number of shortest paths between nodes that a particular node shows up on. Nodes are colored according to their community assignment, as determined by the Leiden algorithm. **(D)** Correlogram indicating the strength of input-output connections between all pairs of regions in the dataset. The color bar indicates the log-normalized projection density between any two regions. Regions are grouped according to their community membership. **(E)** Bar graph comparing projection strength between regions significantly correlated with cilia length vs. those not correlated. Projection strength equals the log normalization of normalized (by injection volume) projection density. **(F)** Fraction of region pairs significantly correlated by cilia length among all regions pairs between two communities. For example, a fraction of 0.111 means that 11.1% of all region pairs between the red and green communities are correlated by cilia length. **(G)** Bar graph comparing projection strength between regions significantly correlated with cilia angle versus those not correlated. **(H)** Fraction of region pairs significantly correlated by cilia angle among all regions pairs between two communities. **(I)** Cilia length correlation network. The spring embedding puts regions more highly correlated closer together. Betweenness and community membership are shown as node size and color, respectively. **(J)** Cilia length correlogram shows the groupings of different regions by community, as well as their correlation patterns. **(K)** Cilia angle correlation network. Betweenness and community membership are shown as node size and color, respectively. **(L)** Cilia angle correlogram shows the groupings of different regions by community, as well as their correlation patterns. Underlying data of Fig 7C-L are available in S2 Data, and from the publicly available Allen Mouse Brain Connectivity Atlas Data.

We next assessed whether fluctuations in cilia length and angle over the 24-h period correlated across different brain regions. Our analysis revealed significant correlations in cilia length changes, with the DMH and VMH showing a particularly strong correlation (*r* = 9.3). Positive correlations were also observed between the PFC and several regions, including the DMH, VMH, LH, AHC, and MC, while CA1 cilia lengths negatively correlated with the DMH and VMH but positively with the RS, NAc, and DG (Fig 7A). Similarly, cilia angle fluctuations exhibited substantial correlations across various brain regions. The strongest correlation was observed between the DMH and VMH (*r* = 0.79), with other notable correlations in hypothalamic regions such as AHC-LH, ARC-PVN, and PVN-SCN. Negative correlations were found between the PVN and VMH, and between cilia angles in the CA1 and TU regions (Fig 7B). We next tested the hypothesis that more highly correlated brain regions, either in cilia length or angle, may be preferentially interconnected. Using the Louvain community detection algorithm on a network representation of the anatomical connections among these regions, we found three primary communities. The first and smallest community (“red”) contained the three hippocampal regions DG, CA1, and CA3. The second community (“green”) contained cortical regions, the striatum, and somewhat surprisingly, the SCN. The third community (“purple”) contained all of the other regions, which overall had less strongly defined interconnectivity relationships (Fig 7D). Based on these data, we then asked if regions that had strong length correlations (*p* < 0.05) were more likely to have stronger interconnectivity than regions with weak length correlations (*p* > 0.05). We found no significant difference in normalized projection strength between pairs of regions correlated with cilia length versus those not correlated (Fig 7E), indicating that projection strength between regions does not influence cilia length. We next examined all pairs of regions within and across different clusters and identified a bias toward regions having correlated cilia lengths if they were present within particular communities; this relationship was most prominent between regions in the red and green communities (Fig 7F). The fraction of correlated pairs between red-green communities (0.111) was in the 9.1st percentile relative to the random distribution, indicating that cilia lengths were more likely than by chance to be correlated between pairs located in red and green communities (Fig 7F).

We next tested for the same relationship between cilia angle and connectivity. As with cilia length, we did not identify a significant difference in projection strength between pairs of regions correlated with cilia angle versus those not correlated (Fig 7G). We then assessed the relationship between community identity and cilia angle correlation and found that the fraction of correlated pairs within the purple community (0.107) was 7.4th percentile relative to the random distribution (Fig 7H). These data together indicate that while the connectivity strength between directly connected pairs did not appear to relate to cilia length or angle, the presence within particular communities did appear to relate to both cilia length and angle, indicating that the network architecture of the brain exhibits some relationship with cilia length and angle correlations across regions.

Given the lack of clear relationship between direct anatomical connectivity and cilia length or angle correlations, we next applied the network approach on the cilia length and angle correlation data directly. In order to do this, we built correlation networks using a log-scaled *R* value that yielded a nicer distribution for edge weights, and again used the Louvain community detection algorithm to assign communities (Fig 7I–7L). These results yielded four communities that did not distinctly track by anatomical connectivity, as evidenced by a different regional distribution across communities. Interestingly, we observed a number of regions that cluster together in both cilia length and angle correlation networks. Notably, NAc, CA1, CA3, TU cluster together for both, as do AHC, PFC, MC, and SSC (Fig 7I–7L). The NAc and TU are both striatal regions while CA1 and CA3 are both hippocampal, and PFC, MC, and SSC are all cortical regions. However, the exact relationship between these sets of regions that co-cluster is not immediately apparent, again suggesting that factors other than direct connectivity likely influence the non-random correlations in cilia length and angle across regions.

## Discussion

In our study, we examined the spatiotemporal patterns of cilia length and orientation across 22 mouse brain regions. Our findings revealed substantial variation in cilia length across these regions, and distinct temporal oscillation in certain regions. We also observed non-random distribution patterns in cilia orientation across the brain, suggesting a structured spatial organization.

To investigate spatiotemporal variability in primary cilia dynamics across mouse brain regions, we developed automated image analysis algorithms for high-throughput analysis of cilia length and orientation. These algorithms enabled the examination of over 10 million individual cilia, creating the largest spatial and temporal atlas of cilia metrics. Automated measurements were validated through manual measurements of cilia length and angles in each of the 22 brain regions examined.

Our study uncovered marked variations in cilia length among different brain regions, with the NAs exhibiting the shortest average cilia length and the TU the longest. Our cilia length findings are in agreement with those reported by Sipos and colleagues across most of the examined regions, though discrepancies were observed within the hypothalamus. Notably, our measurements were based on hundreds of thousands of cilia per region, compared to a maximum of 200 cilia analyzed in the earlier study [57]. Further, while Sipos and colleagues‘s study captured a static snapshot at a single time point, our analysis dynamically tracked cilia length variations every 2 h over a 24-h period [57]. The discrepancy in cilia length within the hypothalamus between the two studies is especially interesting given the profound variability we observed in these regions over the course of the day. This highlights the importance of specifying the timing of the day when reporting cilia-related metrics.

The variations in cilia length across brain regions raise questions about their potential role in functional specialization tailored to the needs of each brain structure, including whether longer cilia might provide a larger surface area for receptor expression, thereby enhancing cellular sensitivity to extracellular signaling molecules. This prompts further investigation of whether the presence of the longest cilia in the TU reflects the critical role of cilia in olfaction [58–61]. Interestingly, the NAc displayed cilia lengths similar to those in the adjacent RS and CS, while the NAs had the shortest cilia lengths among all examined brain regions. This is noteworthy given the anatomical proximity of the TU, RS, CS, and NAc, which form one anatomical cluster, whereas the NAs is classified in a different anatomical cluster [62–65]. Further supporting this anatomical-functional relationship is the strong correlation observed in cilia length across the hypothalamic regions particularly the DMH and VMH, regions known to share several anatomical and functional characteristics.

We observed notable variations in cilia angles, with the MC having the smallest average angle and the Pir the largest. The finding that cilia display distinct orientation patterns across various brain regions presents a novel discovery. The distribution of cilia angles and the dominant orientation toward the medial-ventral quadrants across most regions indicate that cilia orientation in the brain follows specific patterns rather than being random. Notably, there is a remarkable consistency in cilia orientation among anatomically or functionally linked structures. For example, cilia in striatal regions (RS, CS, NAc) and hippocampal areas (CA1, CA3, DG) predominantly exhibit orientations toward 180° and/or 270°. Interestingly, the TU, which is classified as a striatal structure, and the MA, known for its substantial neural connections with the striatum, both exhibit a cilia orientation pattern similar to that of the striatum. Conversely, hypothalamic nuclei, cortical regions, and NAs exhibit wider cilia orientation patterns, yet the majority tend toward the medial-ventral quadrant or align with the axis from 45° to 225°. Our results align with previous findings showing CA1 cilia orient along the apical-basal axis, while cortical cilia orient toward the pial surface [66,67].

Our findings suggest that shared connectivity, reflecting common functional or anatomical characteristics, may shape the pattern of cilia orientation. This supports the idea that cilia orientation distribution across the brain is not random and is associated with the unique functional or anatomical features of interconnected regions. The distinctive cilia orientation patterns across various brain regions may also relate to their specific locations or proximities to the associated ventricles. For example, cilia in the striatum, TU, and hippocampus primarily orient toward the lateral ventricles, whereas those in the hypothalamic nuclei appear to align more with the third ventricle, and in the cortices, cilia orient toward both the ventricles and the subarachnoid space.

Our analysis demonstrated that cilia length and angle fluctuate throughout the day across brain regions. Except for the CS and AHC (*P* = 0.05), all tested regions exhibited time-dependent variations in cilia length, with seven also showing fluctutations in orientation. Although changes in cilia length of 1–2 µm may seem modest, they can result in functional alterations of 10–20% [68–70]. These fluctuations across light and dark phases may reflect varying sensory or signaling needs during these periods. The finding that cilia angles also follow time-dependent fluctuations in certain brain regions is especially interesting, as it suggests that, beyond length adjustments, the direction of signal sensing or transmission by cilia may also shift throughout the day.

Our Bio-Cycle analysis identified significant rhythmicity in cilia length in five brain regions (NAc, ARC, DMH, VMH, and SSC), with no circadian patterns detected in cilia angle. While the reason for this region-specific rhythmicity remains unclear, it is noteworthy that all five regions are closely associated with rhythmic physiological processes. The NAc is vital hub for motivation and reward-seeking behavior, which notably peak during the active phase (dark) and diminish during the inactive phase (light) in nocturnal mice. Notably, GPCRs located on cilia in the NAc are involved in regulating these behaviors, raising the question of whether oscillations in cilia length contribute or respond to the temporal modulation of reward-related processes, especially that cilia are known to play distinct roles in both the acute and long-term responses to reward-inducing drugs [71]. Our recent study demonstrated that many cilia-related transcripts exhibit circadian rhythmicity in the mouse nucleus accumbens and hypothalamus, with notable overrepresentation in the nucleus accumbens compared to the background transcripts. We also found that the rhythmic patterns of cilia transcripts are shifted under conditions of dopamine system modulation, including D2 receptor deletion and cocaine administration [72].

The hypothalamic structures ARC, DMH, and VMH, which exhibit rhythmic oscillations in cilia length, are well known for regulating physiological functions that follow circadian rhythms, including sleep–wake cycles, feeding behavior, energy balance, body temperature regulation, hormone secretion, and stress responses [34–42,73–77].

Cilia in these regions have been implicated in the regulation of rhythmic physiological processes. For example, cilia in the VMH play a crucial role in maintaining energy balance and skeletal homeostasis [78], with their length dynamically responding to metabolic conditions [79]. Notably, shorter cilia are more prevalent under metabolically unfavorable conditions, such as obesity and leptin resistance [79,80]. Evidence suggests that primary cilia on ARC neurons play a crucial role in regulating energy homeostasis by mediating GPCR signaling in response to peripheral hormonal stimuli, ultimately influencing food intake and metabolic balance [81].

It is noteworthy that CSF composition and flow dynamics exhibit time-of-day-dependent variations, which are regulated in part by the beating of motile cilia [82], with highest flow during the night, when animal activity is at its peak [83]. This raises the possibility that primary cilia dynamics may similarly contribute to the regulation of physiological processes with circadian-like patterns. Our findings that cilia length fluctuates in a rhythmic manner within these regions warrant further studies to determine whether these changes are directly driven by circadian mechanisms or are secondary to other rhythmic physiological processes. The SSC is the only cortical structure that showed rhythmic fluctuations in cilia length. This is interesting in light of previous studies showing rhythmic synaptic changes in the SSC [84–87]. While the exact role of these oscillations remains unclear, emerging evidence suggests that primary cilia may play a direct role in circadian regulation. A recent study demonstrated that cilia in a subset SCN neurons, specifically neuromedin S–producing neurons, exhibit rhythmicity in abundance and length, and that genetic ablation of ciliogenesis in these neurons disrupts intercellular coupling, leading to a loss of circadian coherence and an increased susceptibility to external perturbations [88]. Given that we observed rhythmic cilia length fluctuations in multiple hypothalamic regions, it is possible that cilia in other brain areas also play a role in modulating circadian-regulated functions. However, further research is needed to determine whether these oscillations are directly involved in molecular clock mechanisms or reflect secondary effects of circadian-driven processes.

Cilia host numerous receptors like GPCRs and ion channels, crucial for detecting extracellular signals and initiating intracellular responses. Receptors like MC4R, MCHR1, NPY2R, NPY5R, SSTR3, 5HT6, and DRD1 play key roles in rhythmic functions such as sleep-wake cycles and feeding behavior [37,43–47]. The observed fluctuations in cilia length and orientation raise the question of whether these structural changes could influence receptor availability or sensitivity to ligands, thereby modulating signal transduction and time-dependent physiological processes.

One limitation of our study is its exclusive focus on ADCY3-positive cilia, which may overlook the diversity of cilia across brain structures and the full length of cilia. However, ADCY3 is a primary marker for cilia in the mature adult brain, especially in neurons, and cilia labeled with the ADCY3 antibody are often longer than those stained with other markers, such as ARL13 and Sstr3, in many brain regions [57,89], suggesting that our measurements of cilia length likely captured the majority of neuronal cilia and accurately represented their total length. Another limitation of our study is that our study did not identify the specific cell types associated with cilia of various lengths and angles. This work primarily aimed to map the spatiotemporal dynamics of cilia across brain regions, with a focus on changes in ciliary length and orientation throughout the circadian cycle; future research should prioritize examining ADCY3-negative cilia and identifying cell types with cilia that exhibit fluctuations in length and angle. Lastly, when interpreting cilia length and orientation data, it is important to consider potential errors from 2D imaging of 3D structures, as detailed in the supplementary material. However, since these errors are consistent across brain regions due to uniform tissue thickness, they likely do not affect comparative analyses, and thus do not compromise the reliability of the relative measurements.

In conclusion, we presented the largest spatiotemporal atlas of cilia to date, revealing complex regional differences and time-dependent fluctuations in cilia dynamics across the mouse brain. Our findings highlight time-dependent variations in cilia length and orientation, with rhythmic patterns observed in specific brain regions. While the functional implications of these dynamic properties remain to be fully understood, the observed patterns suggest that cilia may contribute to the temporal regulation of physiological processes in a region-specific manner. Future research is needed to elucidate the mechanistic links between cilia dynamics, receptor signaling, and brain function over the light-dark cycle.

## Supporting information

S1 Code Custom-developed software (Program 1 and Program 2) used to perform high-throughput automated measurements of cilia lengths and angles. (ZIP)

S1 Video An instructional video providing a detailed, step-by-step walkthrough of the analysis pipeline. (MP4)

S1 Text Supporting methods, results, and figures (Figs A–G) that provide additional details and analyses related to the main study. (DOCX)

S1 Data Comparison of automated vs. manual measurements of cilia length and angle, and measurement of cilia density across 22 brain regions. (XLSX)

S2 Data Regional and brain-wide average cilia lengths calculated using the section-based average method. (XLSX)

S3 Data Summary of periodic fluctuations in cilia length and angle across 22 brain regions, determined by BioCycle analysis. (XLSX)

## Abbreviations

ADCY3: Adenylate cyclase 3
AHC: Anterior hypothalamic area
ARC: Arcuate nucleus
CA1: Cornu Ammonis 1
CA3: Cornu Ammonis 3
cAMP: cyclic AMP
CS: Caudal striatum
CSF: Cerebrospinal fluid
DNN: deep neural network
DG: Dentate Gyrus
DMH: Dorsomedial hypothalamus
DRD1: Dopamine receptor 1
FDR: False Discovery Rate
GPCRs: G-protein coupled receptors
LH: Lateral hypothalamus
MA: Medial amygdala
MAE: Mean absolute error
MC: Motor cortex
MCH: Melanin-concentrating hormone
MCHR1: Melanin-concentrating hormone receptor 1
MC4R: Melanocortin 4 receptor
MPO: Median preoptic area
NAc: Nucleus accumbens core
NAs: Nucleus accumbens shell
NPY2R: Neuropeptide Y receptor 2
NPY5R: Neuropeptide Y receptor 5
PFA: Paraformaldehyde
PFC: Prefrontal cortex
Pir: Piriform cortex
PVN: Paraventricular nucleus
RS: Rostral striatum
SCN: Suprachiasmatic nucleus
SEM: standard error of the mean
SON: Supraoptic nucleus
SSC: Somatosensory cortex
SSTR3: Somatostatin receptor 3
5HT6: Serotonin receptor 6
TU: Olfactory tubercle
VMH: ventromedial hypothalamus
ZTs: Zeitgeber times

## References

[1] Nachury MV, Mick DU. Establishing and regulating the composition of cilia for signal transduction. Nat Rev Mol Cell Biol. 2019;20(7):389–405. doi: 10.1038/s41580-019-0116-4 30948801 PMC6738346

[2] Liu Z, Tu H, Kang Y, Xue Y, Ma D, Zhao C, et al. Primary cilia regulate hematopoietic stem and progenitor cell specification through Notch signaling in zebrafish. Nat Commun. 2019;10(1):1839. doi: 10.1038/s41467-019-09403-7 31015398 PMC6478842

[3] Guo J, Otis JM, Higginbotham H, Monckton C, Cheng J, Asokan A, et al. Primary cilia signaling shapes the development of interneuronal connectivity. Dev Cell. 2017;42(3):286-300.e4. doi: 10.1016/j.devcel.2017.07.010 28787594 PMC5571900

[4] Nechipurenko IV, Doroquez DB, Sengupta P. Primary cilia and dendritic spines: different but similar signaling compartments. Mol Cells. 2013;36(4):288–303. doi: 10.1007/s10059-013-0246-z 24048681 PMC3837705

[5] Berbari NF, O’Connor AK, Haycraft CJ, Yoder BK. The primary cilium as a complex signaling center. Curr Biol. 2009;19(13):R526-35. doi: 10.1016/j.cub.2009.05.025 19602418 PMC2814769

[6] Wheatley DN. Nanobiology of the primary cilium—paradigm of a multifunctional nanomachine complex. Methods Cell Biol. 2008;90:139–56. doi: 10.1016/S0091-679X(08)00807-8 19195549

[7] Han Y-G, Spassky N, Romaguera-Ros M, Garcia-Verdugo J-M, Aguilar A, Schneider-Maunoury S, et al. Hedgehog signaling and primary cilia are required for the formation of adult neural stem cells. Nat Neurosci. 2008;11(3):277–84. doi: 10.1038/nn2059 18297065

[8] Breunig JJ, Sarkisian MR, Arellano JI, Morozov YM, Ayoub AE, Sojitra S, et al. Primary cilia regulate hippocampal neurogenesis by mediating sonic hedgehog signaling. Proc Natl Acad Sci U S A. 2008;105(35):13127–32. doi: 10.1073/pnas.0804558105 18728187 PMC2529104

[9] Oishi I, Kawakami Y, Raya A, Callol-Massot C, Izpisúa Belmonte JC. Regulation of primary cilia formation and left-right patterning in zebrafish by a noncanonical Wnt signaling mediator, duboraya. Nat Genet. 2006;38(11):1316–22. doi: 10.1038/ng1892 17013396

[10] Kuhara A, Okumura M, Kimata T, Tanizawa Y, Takano R, Kimura KD, et al. Temperature sensing by an olfactory neuron in a circuit controlling behavior of *C. elegans*. Science. 2008;320(5877):803–7. doi: 10.1126/science.1148922 18403676

[11] Humphries S. A physical explanation of the temperature dependence of physiological processes mediated by cilia and flagella. Proc Natl Acad Sci U S A. 2013;110(36):14693–8. doi: 10.1073/pnas.1300891110 23959901 PMC3767513

[12] O’Callaghan C, Achaval M, Forsythe I, Barry PW. Brain and respiratory cilia: the effect of temperature. Biol Neonate. 1995;68(6):394–7. doi: 10.1159/000244261 8721882

[13] Clary-Meinesz CF, Cosson J, Huitorel P, Blaive B. Temperature effect on the ciliary beat frequency of human nasal and tracheal ciliated cells. Biol Cell. 1992;76(3):335–8. doi: 10.1016/0248-4900(92)90436-5 1305479

[14] Nonaka S, Tanaka Y, Okada Y, Takeda S, Harada A, Kanai Y, et al. Randomization of left-right asymmetry due to loss of nodal cilia generating leftward flow of extraembryonic fluid in mice lacking KIF3B motor protein. Cell. 1998;95(6):829–37. doi: 10.1016/s0092-8674(00)81705-5 9865700

[15] Yoshiba S, Shiratori H, Kuo IY, Kawasumi A, Shinohara K, Nonaka S, et al. Cilia at the node of mouse embryos sense fluid flow for left-right determination via Pkd2. Science. 2012;338(6104):226–31. doi: 10.1126/science.1222538 22983710 PMC3711115

[16] Nauli SM, Jin X, AbouAlaiwi WA, El-Jouni W, Su X, Zhou J. Non-motile primary cilia as fluid shear stress mechanosensors. Methods Enzymol. 2013;525:1–20. doi: 10.1016/B978-0-12-397944-5.00001-8 23522462 PMC4096622

[17] McGlashan SR, Knight MM, Chowdhury TT, Joshi P, Jensen CG, Kennedy S, et al. Mechanical loading modulates chondrocyte primary cilia incidence and length. Cell Biol Int. 2010;34(5):441–6. doi: 10.1042/CBI20090094 20100169

[18] Duffy MP, Sup ME, Guo XE. Adenylyl cyclase 3 regulates osteocyte mechanotransduction and primary cilium. Biochem Biophys Res Commun. 2021;573:145–50. doi: 10.1016/j.bbrc.2021.08.033 34411897 PMC8406666

[19] Ou Y, Ruan Y, Cheng M, Moser JJ, Rattner JB, van der Hoorn FA. Adenylate cyclase regulates elongation of mammalian primary cilia. Exp Cell Res. 2009;315(16):2802–17. doi: 10.1016/j.yexcr.2009.06.028 19576885 PMC3161028

[20] Tuxhorn J, Daise T, Dentler WL. Regulation of flagellar length in Chlamydomonas. Cell Motil Cytoskeleton. 1998;40(2):133–46. doi: 10.1002/(SICI)1097-0169(1998)40:2<133::AID-CM3>3.0.CO;2-G 9634211

[21] Brodsky M, Lesiak AJ, Croicu A, Cohenca N, Sullivan JM, Neumaier JF. 5-HT6 receptor blockade regulates primary cilia morphology in striatal neurons. Brain Res. 2017;1660:10–9. doi: 10.1016/j.brainres.2017.01.010 28087224 PMC5392252

[22] Alhassen W, Kobayashi Y, Su J, Robbins B, Nguyen H, Myint T, et al. Regulation of brain primary cilia length by MCH signaling: evidence from pharmacological, genetic, optogenetic, and chemogenic manipulations. Mol Neurobiol. 2022;59(1):245–65. doi: 10.1007/s12035-021-02511-w 34665407 PMC9083846

[23] Abdul-Majeed S, Nauli SM. Dopamine receptor type 5 in the primary cilia has dual chemo- and mechano-sensory roles. Hypertension. 2011;58(2):325–31. doi: 10.1161/HYPERTENSIONAHA.111.172080 21709211 PMC3150550

[24] Upadhyay VS, Muntean BS, Kathem SH, Hwang JJ, Aboualaiwi WA, Nauli SM. Roles of dopamine receptor on chemosensory and mechanosensory primary cilia in renal epithelial cells. Front Physiol. 2014;5:72. doi: 10.3389/fphys.2014.00072 24616705 PMC3935400

[25] Christensen ST, Pedersen SF, Satir P, Veland IR, Schneider L. The primary cilium coordinates signaling pathways in cell cycle control and migration during development and tissue repair. Curr Top Dev Biol. 2008;85:261–301. doi: 10.1016/S0070-2153(08)00810-7 19147009

[26] Jones TJ, Adapala RK, Geldenhuys WJ, Bursley C, AbouAlaiwi WA, Nauli SM, et al. Primary cilia regulates the directional migration and barrier integrity of endothelial cells through the modulation of hsp27 dependent actin cytoskeletal organization. J Cell Physiol. 2012;227(1):70–6. doi: 10.1002/jcp.22704 21837772 PMC3202021

[27] Schneider L, Cammer M, Lehman J, Nielsen SK, Guerra CF, Veland IR, et al. Directional cell migration and chemotaxis in wound healing response to PDGF-AA are coordinated by the primary cilium in fibroblasts. Cell Physiol Biochem. 2010;25(2–3):279–92. doi: 10.1159/000276562 20110689 PMC2924811

[28] Dewan K, Benloucif S, Reid K, Wolfe LF, Zee PC. Light-induced changes of the circadian clock of humans: increasing duration is more effective than increasing light intensity. Sleep. 2011;34(5):593–9. doi: 10.1093/sleep/34.5.593 21532952 PMC3079938

[29] Wright KP Jr, McHill AW, Birks BR, Griffin BR, Rusterholz T, Chinoy ED. Entrainment of the human circadian clock to the natural light-dark cycle. Curr Biol. 2013;23(16):1554–8. doi: 10.1016/j.cub.2013.06.039 23910656 PMC4020279

[30] Damiola F, Le Minh N, Preitner N, Kornmann B, Fleury-Olela F, Schibler U. Restricted feeding uncouples circadian oscillators in peripheral tissues from the central pacemaker in the suprachiasmatic nucleus. Genes Dev. 2000;14(23):2950–61. doi: 10.1101/gad.183500 11114885 PMC317100

[31] Buhr ED, Yoo S-H, Takahashi JS. Temperature as a universal resetting cue for mammalian circadian oscillators. Science. 2010;330(6002):379–85. doi: 10.1126/science.1195262 20947768 PMC3625727

[32] Casey T, Patel OV, Plaut K. Transcriptomes reveal alterations in gravity impact circadian clocks and activate mechanotransduction pathways with adaptation through epigenetic change. Physiol Genomics. 2015;47(4):113–28. doi: 10.1152/physiolgenomics.00117.2014 25649141

[33] Robinson EL, Fuller CA. Gravity and thermoregulation: metabolic changes and circadian rhythms. Pflugers Arch. 2000;441(2-3 Suppl):R32-8. doi: 10.1007/s004240000329 11200977

[34] Fuller CA. The effects of gravity on the circadian timing system. J Gravit Physiol. 1994;1(1):P1-4. 11538728

[35] Gu W, Geddes BJ, Zhang C, Foley KP, Stricker-Krongrad A. The prolactin-releasing peptide receptor (GPR10) regulates body weight homeostasis in mice. J Mol Neurosci. 2004;22(1–2):93–103. doi: 10.1385/JMN:22:1-2:93 14742914

[36] Nagata A, Hamamoto A, Horikawa M, Yoshimura K, Takeda S, Saito Y. Characterization of ciliary targeting sequence of rat melanin-concentrating hormone receptor 1. Gen Comp Endocrinol. 2013;188:159–65. doi: 10.1016/j.ygcen.2013.02.020 23467069

[37] Omori Y, Chaya T, Yoshida S, Irie S, Tsujii T, Furukawa T. Identification of G Protein-Coupled Receptors (GPCRs) in primary cilia and their possible involvement in body weight control. PLoS One. 2015;10(6):e0128422. doi: 10.1371/journal.pone.0128422 26053317 PMC4459993

[38] Hughes JW, Cho JH, Conway HE, DiGruccio MR, Ng XW, Roseman HF, et al. Primary cilia control glucose homeostasis via islet paracrine interactions. Proc Natl Acad Sci U S A. 2020;117(16):8912–23. doi: 10.1073/pnas.2001936117 32253320 PMC7184063

[39] Lee H, Song J, Jung JH, Ko HW. Primary cilia in energy balance signaling and metabolic disorder. BMB Rep. 2015;48(12):647–54. doi: 10.5483/bmbrep.2015.48.12.229 26538252 PMC4791320

[40] Berbari NF, Pasek RC, Malarkey EB, Yazdi SMZ, McNair AD, Lewis WR, et al. Leptin resistance is a secondary consequence of the obesity in ciliopathy mutant mice. Proc Natl Acad Sci U S A. 2013;110(19):7796–801. doi: 10.1073/pnas.1210192110 23599282 PMC3651481

[41] Koemeter-Cox AI, Sherwood TW, Green JA, Steiner RA, Berbari NF, Yoder BK, et al. Primary cilia enhance kisspeptin receptor signaling on gonadotropin-releasing hormone neurons. Proc Natl Acad Sci U S A. 2014;111(28):10335–40. doi: 10.1073/pnas.1403286111 24982149 PMC4104922

[42] Diniz GB, Battagello DS, Klein MO, Bono BSM, Ferreira JGP, Motta-Teixeira LC, et al. Ciliary melanin-concentrating hormone receptor 1 (MCHR1) is widely distributed in the murine CNS in a sex-independent manner. J Neurosci Res. 2020;98(10):2045–71. doi: 10.1002/jnr.24651 32530066

[43] Brailov I, Bancila M, Brisorgueil MJ, Miquel MC, Hamon M, Vergé D. Localization of 5-HT(6) receptors at the plasma membrane of neuronal cilia in the rat brain. Brain Res. 2000;872(1–2):271–5. doi: 10.1016/s0006-8993(00)02519-1 10924708

[44] Berbari NF, Lewis JS, Bishop GA, Askwith CC, Mykytyn K. Bardet-Biedl syndrome proteins are required for the localization of G protein-coupled receptors to primary cilia. Proc Natl Acad Sci U S A. 2008;105(11):4242–6. doi: 10.1073/pnas.0711027105 18334641 PMC2393805

[45] Domire JS, Green JA, Lee KG, Johnson AD, Askwith CC, Mykytyn K. Dopamine receptor 1 localizes to neuronal cilia in a dynamic process that requires the Bardet-Biedl syndrome proteins. Cell Mol Life Sci. 2011;68(17):2951–60. doi: 10.1007/s00018-010-0603-4 21152952 PMC3368249

[46] Händel M, Schulz S, Stanarius A, Schreff M, Erdtmann-Vourliotis M, Schmidt H, et al. Selective targeting of somatostatin receptor 3 to neuronal cilia. Neuroscience. 1999;89(3):909–26. doi: 10.1016/s0306-4522(98)00354-6 10199624

[47] Varela L, Horvath TL. Neuronal cilia: another player in the melanocortin system. Trends Mol Med. 2018;24(4):333–4. doi: 10.1016/j.molmed.2018.02.004 29501261

[48] Chen S, Alhassen W, Vakil Monfared R, Vachirakorntong B, Nauli SM, Baldi P, et al. Dynamic changes of brain cilia transcriptomes across the human lifespan. Int J Mol Sci. 2021;22(19):10387. doi: 10.3390/ijms221910387 34638726 PMC8509004

[49] Baldi P, Alhassen W, Chen S, Nguyen H, Khoudari M, Alachkar A. Large-scale analysis reveals spatiotemporal circadian patterns of cilia transcriptomes in the primate brain. J Neurosci Res. 2021;99(10):2610–24. doi: 10.1002/jnr.24919 34310750 PMC11391745

[50] Alhassen W, Chen S, Vawter M, Robbins BK, Nguyen H, Myint TN, et al. Patterns of cilia gene dysregulations in major psychiatric disorders. Prog Neuropsychopharmacol Biol Psychiatry. 2021;109:110255. doi: 10.1016/j.pnpbp.2021.110255 33508383 PMC9121176

[51] Oh SW, Harris JA, Ng L, Winslow B, Cain N, Mihalas S, et al. A mesoscale connectome of the mouse brain. Nature. 2014;508(7495):207–14. doi: 10.1038/nature13186 24695228 PMC5102064

[52] Muller B, Richards AJ, Jin B, Lu X. GOGrapher: a Python library for GO graph representation and analysis. BMC Res Notes. 2009;2:122. doi: 10.1186/1756-0500-2-122 19583843 PMC2714316

[53] Hagberg AA. Exploring network structure, dynamics, and function using NetworkX. In: Proceedings of the 7th Python in Science Conference, Pasadena, CA, USA, 2008.

[54] Sievert C. Interactive web-based data visualization with R, plotly, and shiny. 2020.

[55] Agostinelli F, Ceglia N, Shahbaba B, Sassone-Corsi P, Baldi P. What time is it? Deep learning approaches for circadian rhythms. Bioinformatics. 2016;32(19):3051. doi: 10.1093/bioinformatics/btw504 27542773 PMC5039929

[56] Agostinelli F, Ceglia N, Shahbaba B, Sassone-Corsi P, Baldi P. What time is it? Deep learning approaches for circadian rhythms. Bioinformatics. 2016;32(12):i8–17. doi: 10.1093/bioinformatics/btw243 27307647 PMC4908327

[57] Sipos É, Komoly S, Ács P. Quantitative comparison of primary cilia marker expression and length in the mouse brain. J Mol Neurosci. 2018;64(3):397–409. doi: 10.1007/s12031-018-1036-z 29464516

[58] Joiner AM, Green WW, McIntyre JC, Allen BL, Schwob JE, Martens JR. Primary cilia on horizontal basal cells regulate regeneration of the olfactory epithelium. J Neurosci. 2015;35(40):13761–72. doi: 10.1523/JNEUROSCI.1708-15.2015 26446227 PMC4595624

[59] Pifferi S, Menini A, Kurahashi T. Signal transduction in vertebrate olfactory cilia. In: Menini A, Editor. The neurobiology of olfaction. Boca Raton (FL): Frontiers in Neuroscience. 2010. 21882437

[60] Challis RC, Tian H, Wang J, He J, Jiang J, Chen X, et al. An olfactory cilia pattern in the mammalian nose ensures high sensitivity to odors. Curr Biol. 2015;25(19):2503–12. doi: 10.1016/j.cub.2015.07.065 26365258 PMC4596779

[61] Qiu L, LeBel RP, Storm DR, Chen X. Type 3 adenylyl cyclase: a key enzyme mediating the cAMP signaling in neuronal cilia. Int J Physiol Pathophysiol Pharmacol. 2016;8(3):95–108. 27785336 PMC5078481

[62] Berendse HW, Groenewegen HJ. Organization of the thalamostriatal projections in the rat, with special emphasis on the ventral striatum. J Comp Neurol. 1990;299(2):187–228. doi: 10.1002/cne.902990206 2172326

[63] Zahm DS, Brog JS. On the significance of subterritories in the “accumbens” part of the rat ventral striatum. Neuroscience. 1992;50(4):751–67. doi: 10.1016/0306-4522(92)90202-d 1448200

[64] Siciliano CA, Calipari ES, Jones SR. Amphetamine potency varies with dopamine uptake rate across striatal subregions. J Neurochem. 2014;131(3):348–55. doi: 10.1111/jnc.12808 24988947 PMC4205180

[65] Shin R, Qin M, Liu Z-H, Ikemoto S. Intracranial self-administration of MDMA into the ventral striatum of the rat: differential roles of the nucleus accumbens shell, core, and olfactory tubercle. Psychopharmacology (Berl). 2008;198(2):261–70. doi: 10.1007/s00213-008-1131-x 18389222 PMC2572734

[66] Sheu S-H, Upadhyayula S, Dupuy V, Pang S, Deng F, Wan J, et al. A serotonergic axon-cilium synapse drives nuclear signaling to alter chromatin accessibility. Cell. 2022;185(18):3390-3407.e18. doi: 10.1016/j.cell.2022.07.026 36055200 PMC9789380

[67] Kirschen GW, Liu H, Lang T, Liang X, Ge S, Xiong Q. The radial organization of neuronal primary cilia is acutely disrupted by seizure and ischemic brain injury. Front Biol (Beijing). 2017;12(2):124–38. doi: 10.1007/s11515-017-1447-1 28473847 PMC5412953

[68] Sherpa RT, Mohieldin AM, Pala R, Wachten D, Ostrom RS, Nauli SM. Sensory primary cilium is a responsive cAMP microdomain in renal epithelia. Sci Rep. 2019;9(1):6523. doi: 10.1038/s41598-019-43002-2 31024067 PMC6484033

[69] Sherpa RT, Atkinson KF, Ferreira VP, Nauli SM. Rapamycin increases length and mechanosensory function of primary cilia in renal epithelial and vascular endothelial cells. Int Educ Res J. 2016;2(12):91–7. 28529994 PMC5436805

[70] Abdul-Majeed S, Moloney BC, Nauli SM. Mechanisms regulating cilia growth and cilia function in endothelial cells. Cell Mol Life Sci. 2012;69(1):165–73. doi: 10.1007/s00018-011-0744-0 21671118 PMC11115144

[71] Ramos C, Roberts JB, Jasso KR, Ten Eyck TW, Everett T, Pozo P, et al. Neuron-specific cilia loss differentially alters locomotor responses to amphetamine in mice. J Neurosci Res. 2021;99(3):827–42. doi: 10.1002/jnr.24755 33175436 PMC8138950

[72] Chen K, Ashtiani KC, Monfared RV, Baldi P, Alachkar A. Circadian cilia transcriptome in mouse brain across physiological and pathological states. Mol Brain. 2024;17(1):67. doi: 10.1186/s13041-024-01143-0 39304885 PMC11414107

[73] Abe M, Herzog ED, Yamazaki S, Straume M, Tei H, Sakaki Y, et al. Circadian rhythms in isolated brain regions. J Neurosci. 2002;22(1):350–6. doi: 10.1523/JNEUROSCI.22-01-00350.2002 11756518 PMC6757616

[74] Myers MG Jr, Olson DP. Central nervous system control of metabolism. Nature. 2012;491(7424):357–63. doi: 10.1038/nature11705 23151578

[75] Chou TC, Bjorkum AA, Gaus SE, Lu J, Scammell TE, Saper CB. Afferents to the ventrolateral preoptic nucleus. J Neurosci. 2002;22(3):977–90. doi: 10.1523/JNEUROSCI.22-03-00977.2002 11826126 PMC6758527

[76] Santoso P, Nakata M, Ueta Y, Yada T. Suprachiasmatic vasopressin to paraventricular oxytocin neurocircuit in the hypothalamus relays light reception to inhibit feeding behavior. Am J Physiol Endocrinol Metab. 2018;315(4):E478–88. doi: 10.1152/ajpendo.00338.2016 28174180

[77] Acosta-Galvan G, Yi C-X, van der Vliet J, Jhamandas JH, Panula P, Angeles-Castellanos M, et al. Interaction between hypothalamic dorsomedial nucleus and the suprachiasmatic nucleus determines intensity of food anticipatory behavior. Proc Natl Acad Sci U S A. 2011;108(14):5813–8. doi: 10.1073/pnas.1015551108 21402951 PMC3078408

[78] Sun JS, Yang DJ, Kinyua AW, Yoon SG, Seong JK, Kim J, et al. Ventromedial hypothalamic primary cilia control energy and skeletal homeostasis. J Clin Invest. 2021;131(1):e138107. doi: 10.1172/JCI138107 33021968 PMC7773357

[79] Han YM, Kang GM, Byun K, Ko HW, Kim J, Shin M-S, et al. Leptin-promoted cilia assembly is critical for normal energy balance. J Clin Invest. 2014;124(5):2193–7. doi: 10.1172/JCI69395 24667636 PMC4001529

[80] Kang GM, Han YM, Ko HW, Kim J, Oh BC, Kwon I, et al. Leptin elongates hypothalamic neuronal cilia via transcriptional regulation and actin destabilization. J Biol Chem. 2015;290(29):18146–55. doi: 10.1074/jbc.M115.639468 26041775 PMC4505059

[81] Loktev AV, Jackson PK. Neuropeptide Y family receptors traffic via the Bardet-Biedl syndrome pathway to signal in neuronal primary cilia. Cell Rep. 2013;5(5):1316–29. doi: 10.1016/j.celrep.2013.11.011 24316073

[82] Faubel R, Westendorf C, Bodenschatz E, Eichele G. Cilia-based flow network in the brain ventricles. Science. 2016;353(6295):176–8. doi: 10.1126/science.aae0450 27387952

[83] Hablitz LM, Plá V, Giannetto M, Vinitsky HS, Stæger FF, Metcalfe T, et al. Circadian control of brain glymphatic and lymphatic fluid flow. Nat Commun. 2020;11(1):4411. doi: 10.1038/s41467-020-18115-2 32879313 PMC7468152

[84] Jasinska M, Woznicka O, Jasek-Gajda E, Lis GJ, Pyza E, Litwin JA. Circadian changes of dendritic spine geometry in mouse barrel cortex. Front Neurosci. 2020;14:578881. doi: 10.3389/fnins.2020.578881 33117123 PMC7550732

[85] Jasinska M, Jasek-Gajda E, Woznicka O, Lis GJ, Pyza E, Litwin JA. Circadian clock regulates the shape and content of dendritic spines in mouse barrel cortex. PLoS One. 2019;14(11):e0225394. doi: 10.1371/journal.pone.0225394 31730670 PMC6857954

[86] Jasinska M, Grzegorczyk A, Woznicka O, Jasek E, Kossut M, Barbacka-Surowiak G, et al. Circadian rhythmicity of synapses in mouse somatosensory cortex. Eur J Neurosci. 2015;42(8):2585–94. doi: 10.1111/ejn.13045 26274013

[87] Jasinska M, Grzegorczyk A, Jasek E, Litwin JA, Kossut M, Barbacka-Surowiak G, et al. Daily rhythm of synapse turnover in mouse somatosensory cortex. Acta Neurobiol Exp (Wars). 2014;74(1):104–10. doi: 10.55782/ane-2014-1977 24718049

[88] Tu H-Q, Li S, Xu Y-L, Zhang Y-C, Li P-Y, Liang L-Y, et al. Rhythmic cilia changes support SCN neuron coherence in circadian clock. Science. 2023;380(6648):972–9. doi: 10.1126/science.abm1962 37262147

[89] Brewer KK, Brewer KM, Terry TT, Caspary T, Vaisse C, Berbari NF. Postnatal dynamic ciliary ARL13B and ADCY3 localization in the mouse brain. Cells. 2024;13(3):259. doi: 10.3390/cells13030259 38334651 PMC10854790

