# Supplementary figures and images for: Cilia in the brain display region-dependent oscillations of length and orientation
Source: PLoS Biol. 2025 Jul 11;23(7):e3003197. doi: 10.1371/journal.pbio.3003197 (PMC12250621; doi:10.1371/journal.pbio.3003197)

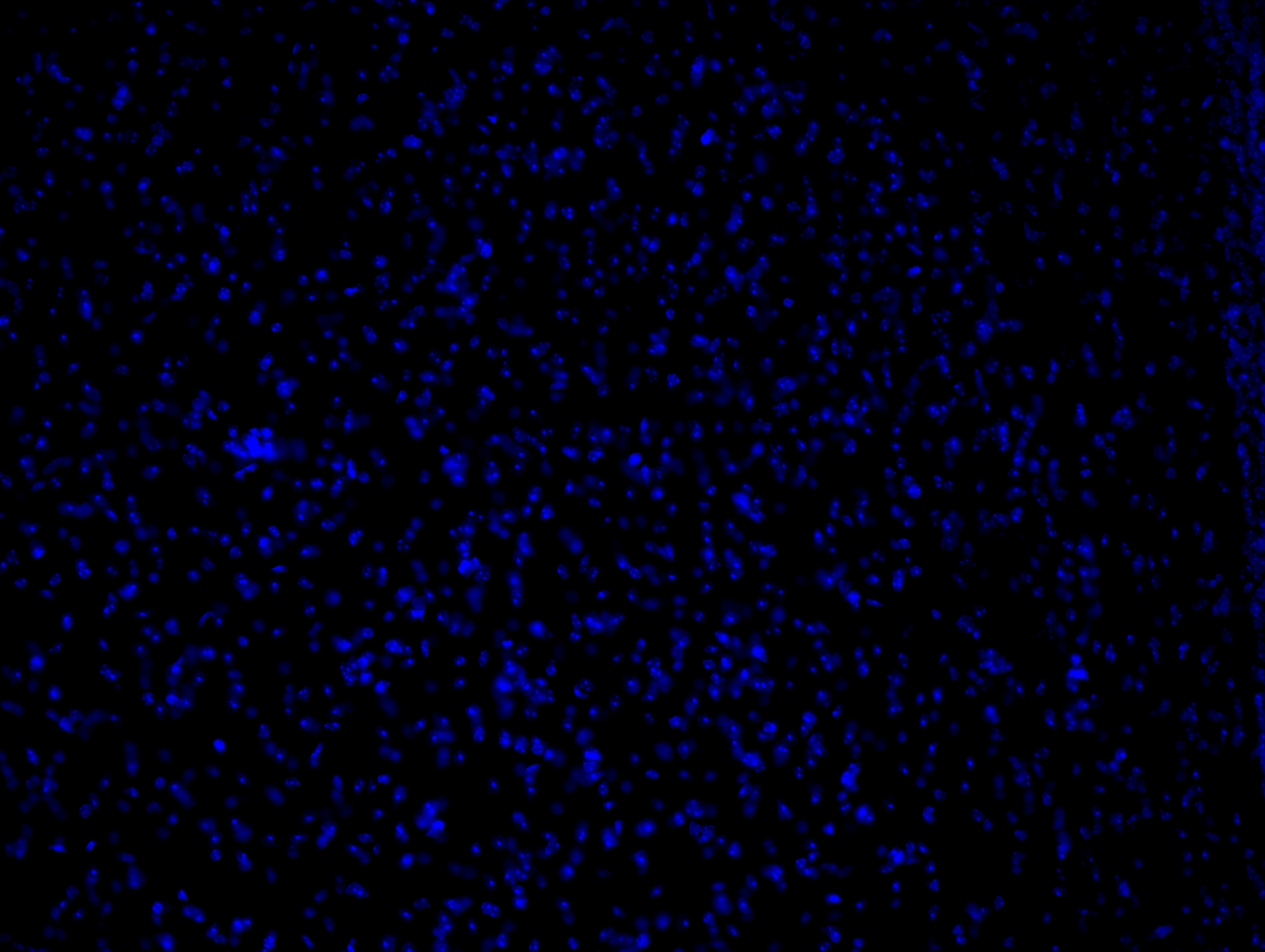

Supplement: S1 Code — (ZIP) [file pbio.3003197.s001.zip › code_for_paper_v2/sample_dapi_dataset/AHC/L2/AHC_L2_LEFT_R00C00_CH3.TIF]

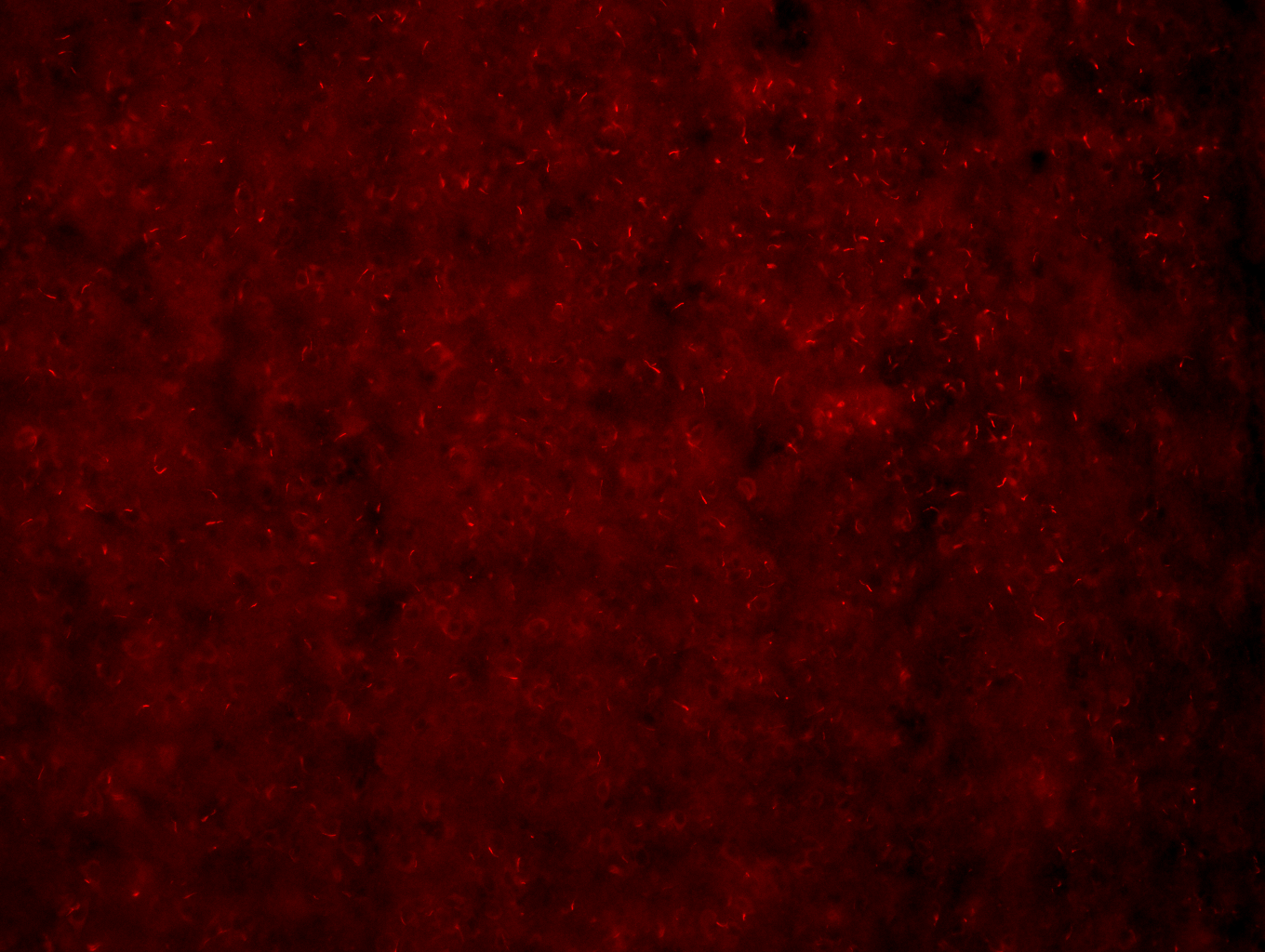

Supplement: S1 Code — (ZIP) [file pbio.3003197.s001.zip › code_for_paper_v2/sample_dapi_dataset/AHC/L2/AHC_L2_LEFT_R00C00_CH2.TIF]

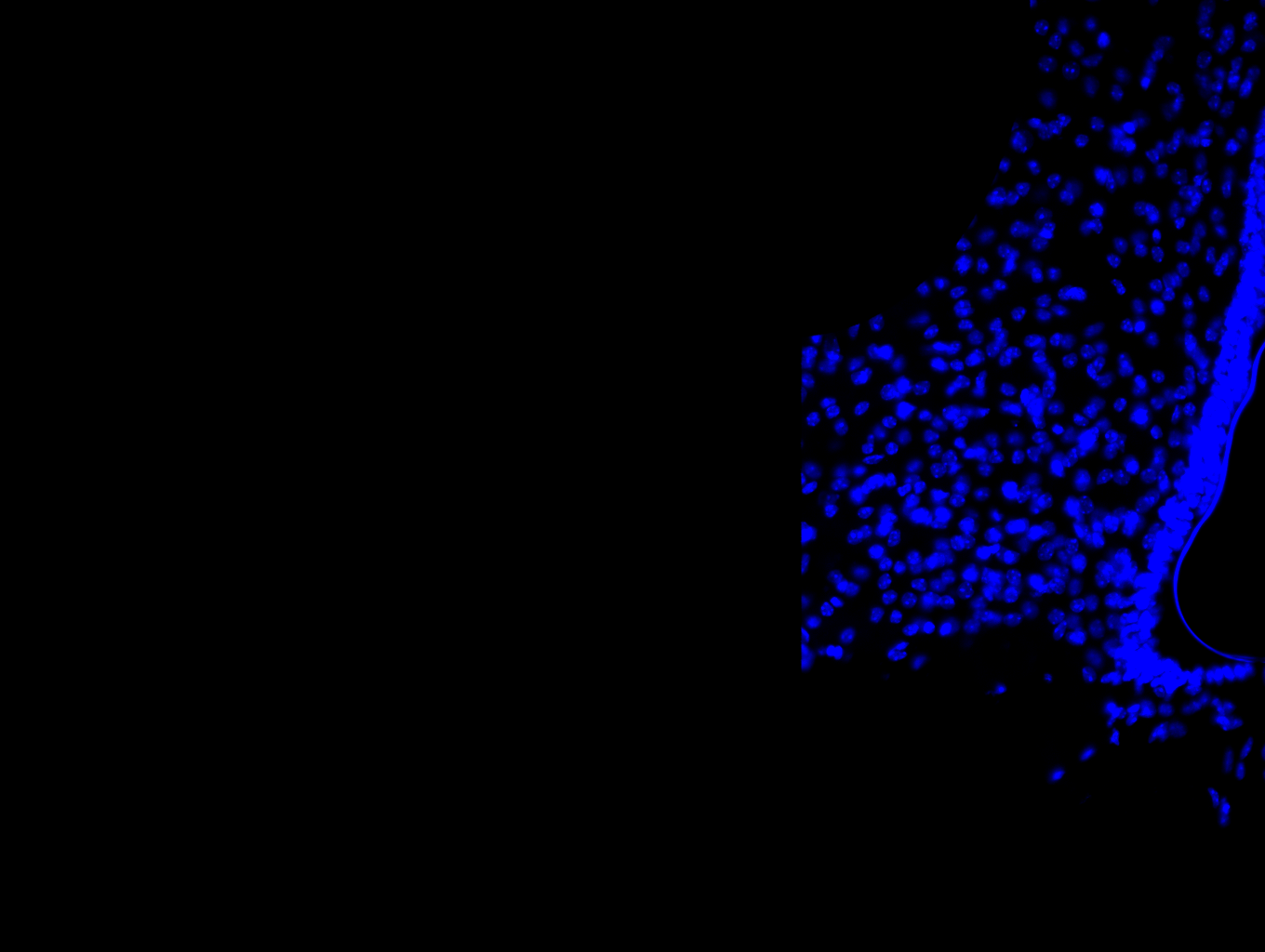

Supplement: S1 Code — (ZIP) [file pbio.3003197.s001.zip › code_for_paper_v2/sample_dapi_dataset/Arc/L2/ARC_L2_LEFT_R00C00_CH3.TIF]

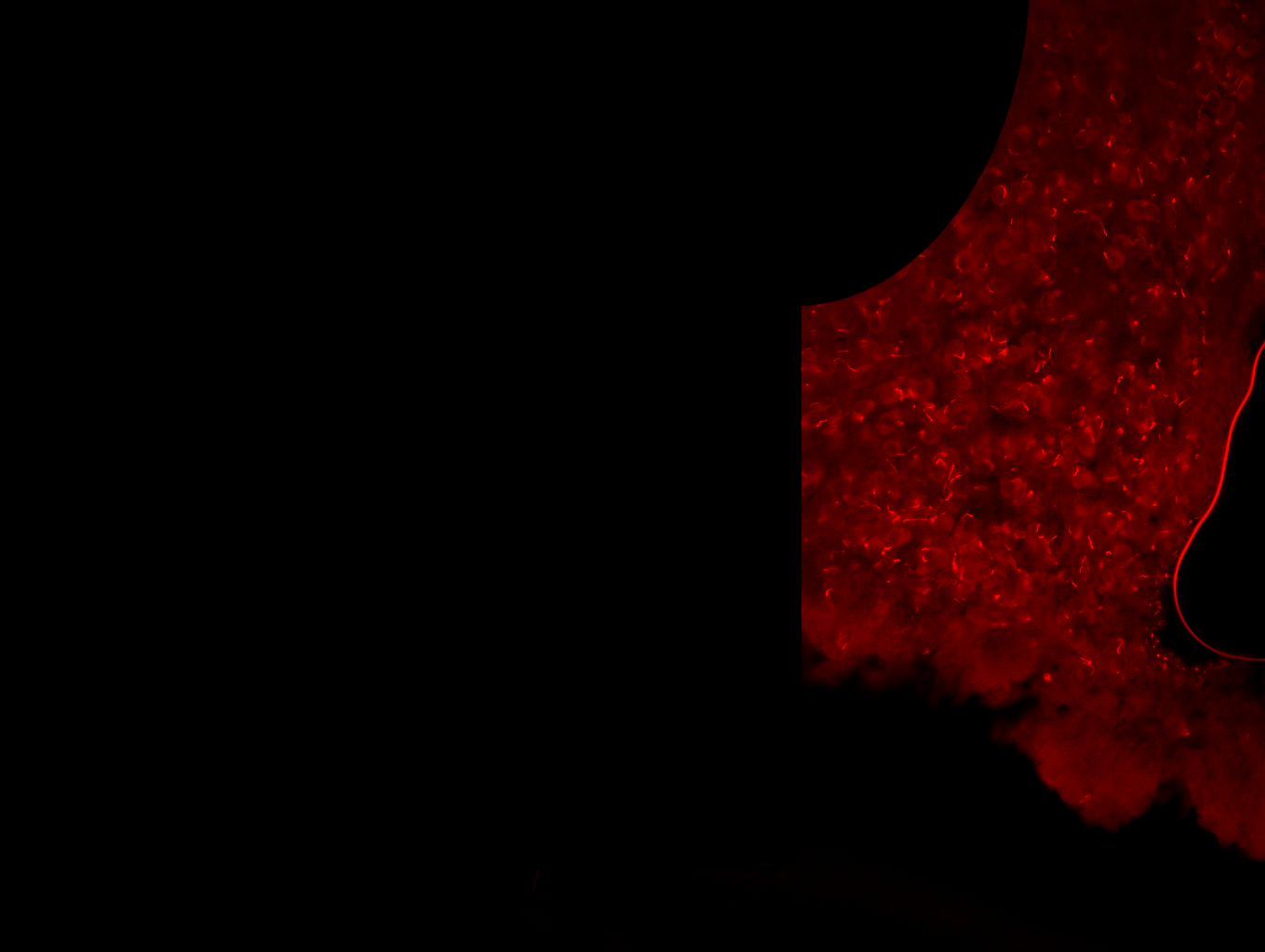

Supplement: S1 Code — (ZIP) [file pbio.3003197.s001.zip › code_for_paper_v2/sample_dapi_dataset/Arc/L2/ARC_L2_LEFT_R00C00_CH2.TIF]

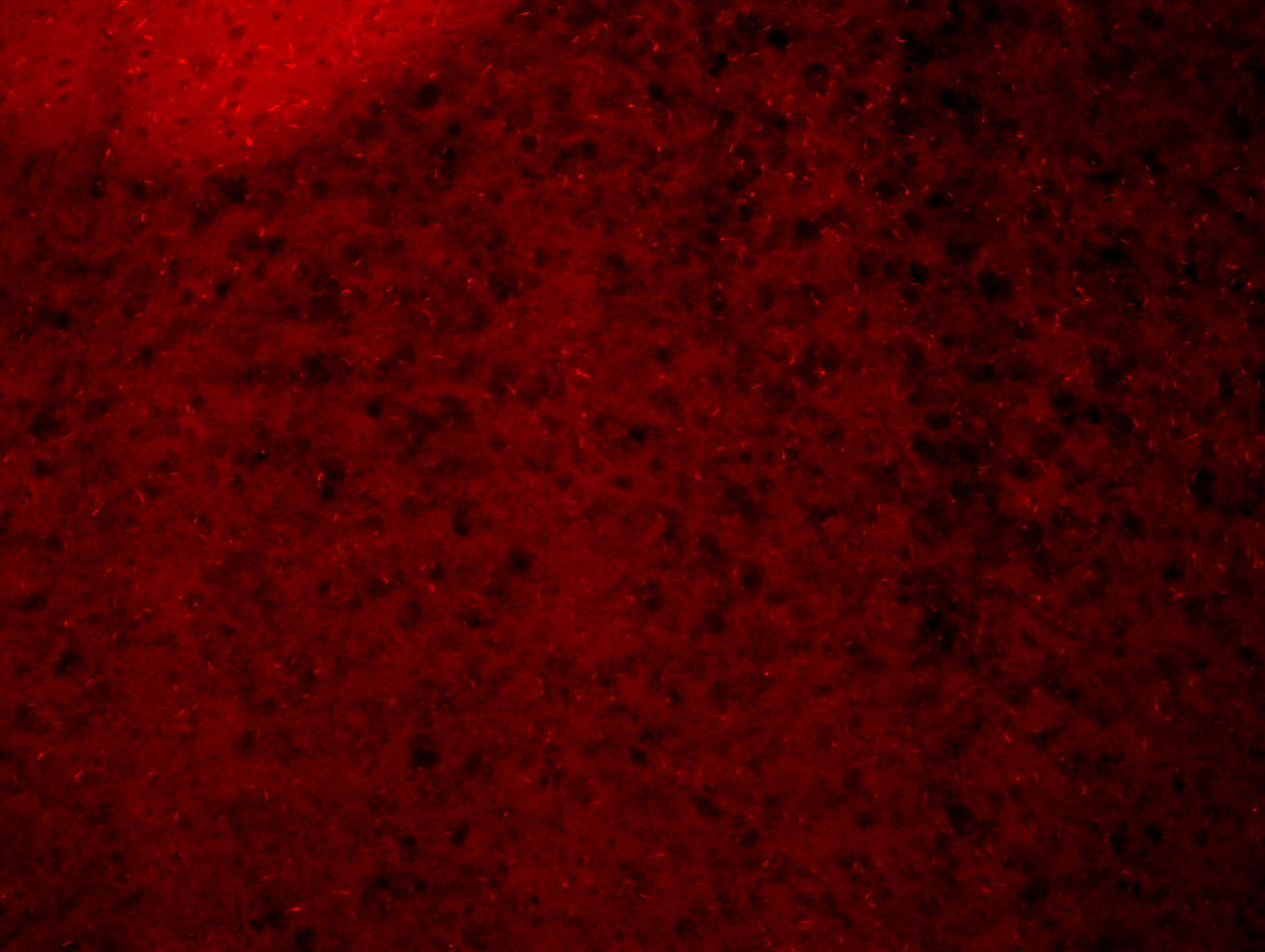

Supplement: S1 Code — (ZIP) [file pbio.3003197.s001.zip › code_for_paper_v2/sample_cilia_dataset/images/AHC/A/Brain 1/AHC_A1_1_LEFT_R00C00_CH2.TIF]

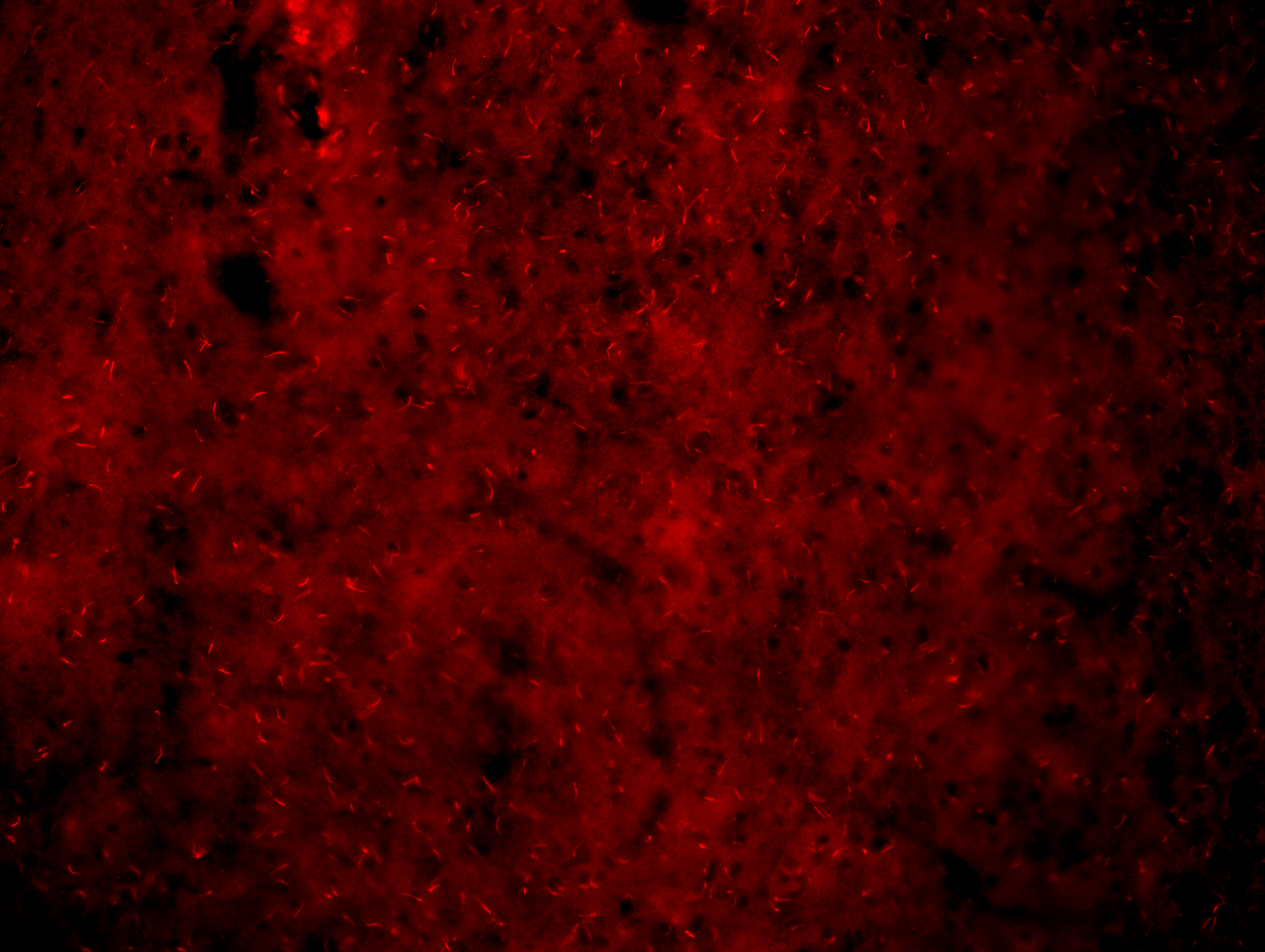

Supplement: S1 Code — (ZIP) [file pbio.3003197.s001.zip › code_for_paper_v2/sample_cilia_dataset/images/AHC/B/Brain 1/AHC_B1_1_LEFT_R00C00_CH2.TIF]

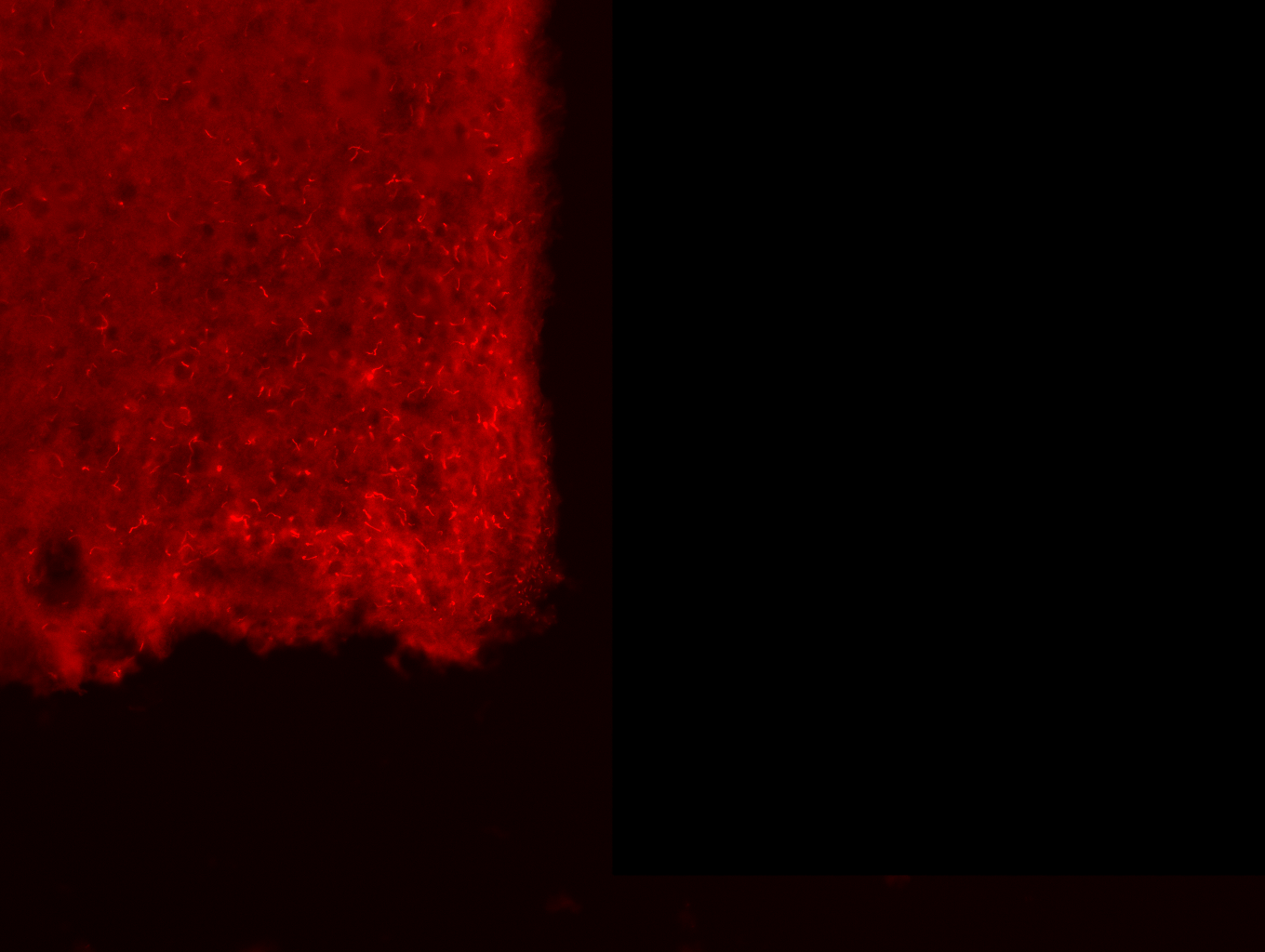

Supplement: S1 Code — (ZIP) [file pbio.3003197.s001.zip › code_for_paper_v2/sample_cilia_dataset/images/ARC/A/BRAIN 1/ARC_A1_1_Left.TIF]

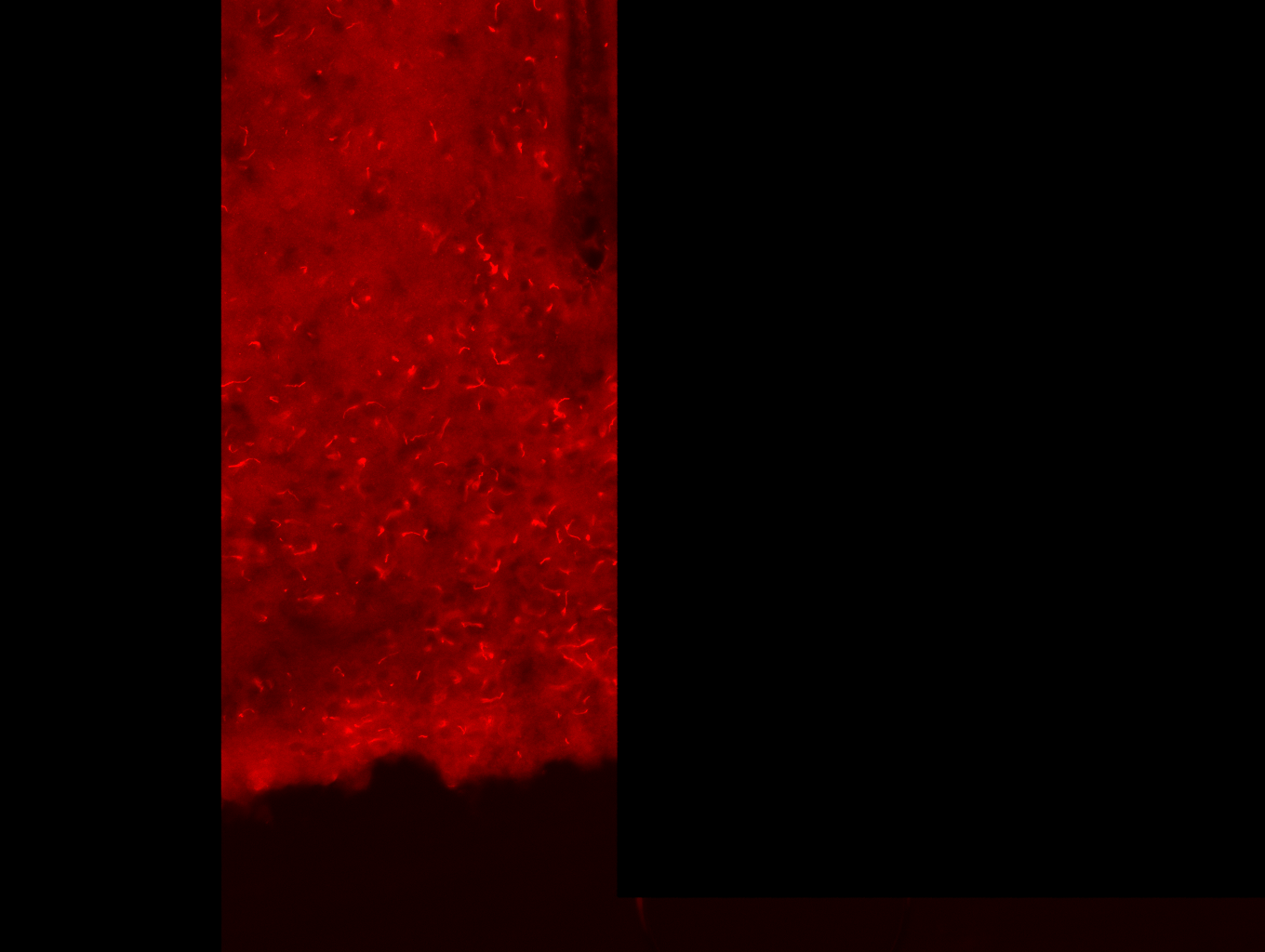

Supplement: S1 Code — (ZIP) [file pbio.3003197.s001.zip › code_for_paper_v2/sample_cilia_dataset/images/ARC/B/BRAIN 1/ARC_B1_1_LEFT.TIF]
