# Supplementary material for: Cilia in the brain display region-dependent oscillations of length and orientation
Source: PLoS Biol. 2025 Jul 11;23(7):e3003197. doi: 10.1371/journal.pbio.3003197 (PMC12250621; doi:10.1371/journal.pbio.3003197)
Supplement: S1 Text — (DOCX) [file pbio.3003197.s003.docx]

**Supplementary Materials**

**Cilia in the brain display region-dependent oscillations of length and orientation**

Roudabeh Vakil Monfared^1a^, Sherif Abdelkarim^2a^, Pieter Derdeyn^3^, Kiki Chen^1^, Hanting Wu^1^, Kenneth Leong^1^, Tiffany Chang^1^, Justine Lee^1^, Sara Versales^1^, Surya M. Nauli^4^, Kevin Beier^1,5-7^,

Pierre Baldi^2,8,9^*, Amal Alachkar^1,8,9^*

1 Department of Pharmaceutical Sciences, School of Pharmacy and Pharmaceutical Sciences, University of California-Irvine, USA

2 Department of Computer Science, School of Information and Computer Sciences, University of California-Irvine, USA

3 Mathematical, Computational, and Systems Biology Program, University of California, Irvine, USA

4 Department of Biomedical and Pharmaceutical Sciences, School of Pharmacy, Chapman University, Health Science Campus, Chapman University, Irvine, USA

5 Department of Physiology and Biophysics, School of medicine, University of California, Irvine, USA

6 Department of Neurobiology and Behavior, University of California, Irvine, CA, USA 92697-4560

7 Department of Biomedical Engineering, University of California, Irvine, CA, USA 92697-4560

8 Center for the Neurobiology of Learning and Memory, University of California, Irvine, Irvine, CA, USA 92697

9 Institute for Genomics and Bioinformatics, School of Information and Computer Sciences, University of California-Irvine, USA

^a^ These authors contributed equally to this work

*** Corresponding Authors**:

- Amal Alachkar

Department of Pharmaceutical Sciences

University of California, Irvine

356A Med Surge II

Irvine CA, 92697-4625

Email: aalachka@uci.edu

- Pierre Baldi, PhD

Departments of Computer Science

School of Information and Computer Sciences

University of California, Irvine

Irvine CA, 92697

Email: pfbaldi@uci.edu

**Supplementary Methods and Results**

**Estimation of instrumental error:**

***Instrumental error:***

The instrumental error in measuring the length of cilia arises from the fact that we are measuring a 3D structure in a 2D plane. This error can be theoretically estimated using basic geometric principles, specifically the Pythagorean theorem.

#### **Geometric considerations**

Assume:

- ***a*** represent the measured 2D length of the cilium.
- ***b*** represent the tissue thickness, which is the dimension not visible in the 2D image.
- ***c*** represent the actual 3D length of the cilium.

In order to understand the potential errors introduced by projecting a 3D structure (a cilium) onto a 2D plane, we need to consider the geometric relationships involved.

Figure A illustrates the best-case scenario, where the measured 2D length (a) is equal to the actual 3D length (c). In this scenario, although the tissue thickness (b) is present and the same as in Figure 2, the cilium is perfectly aligned with the imaging plane, with no angle along the z-axis. As a result, the entire length of the cilium lies along the x-y plane, and the 2D measurement directly reflects the true length.

- **Key point:** In this case, the relationship c=a holds because the cilium is oriented parallel to the imaging plane. The tissue thickness does not contribute to any projection error since there is no hidden length along the z-axis. This represents the ideal situation where the measured 2D length accurately corresponds to the actual 3D length, resulting in zero error.

Figure B shows the worst-case scenario, where the tissue thickness (b) is the same as in Figure 1, but the cilium is angled relative to the imaging plane. In this situation, part of the cilium's length is oriented along the z-axis, which is not visible in the 2D image. As a result, the measured 2D length (a) underestimates the actual 3D length (c), leading to a significant projection error. This maximum projection error is the focus of our calculation.

According to the Pythagorean theorem, the relationship between these quantities is:

$c=\sqrt{a^{2}+b^{2}}$

#### **Instrumental error calculation**

The instrumental error represents the potential underestimation of the cilium’s length due to the 2D projection. It is defined as the difference between the actual 3D length c and the measured 2D length a, expressed as a percentage of the actual length:

$$Instrumental Error (\%)=\left( \frac{c-a}{c} \right) \times100\%$$

Substituting the expression for *c* from the Pythagorean theorem:

$$Instrumental Error (\%)=\left( \frac{\sqrt{a^{2}+b^{2}}-a}{\sqrt{a^{2}+b^{2}}} \right) \times100\%$$

This equation provides a theoretical estimation of the upper limit of the error that could occur due to measuring the cilia in 2D instead of 3D.

***Applying a lower limit on a to reduce errors***

To reduce the potential measurement errors, we impose a lower limit on the measured 2D length a. Specifically, we filter out any cilia measurements where *a* is less than 3 microns. This filtering helps ensure that we filter out vertically oriented cilia as much as possible.

***Limiting the error based on maximum cilia length***

Given that the maximum actual cilia length (cc) is assumed to be 10 microns, this places a natural limit on the possible projection error. For example, if the measured 2D length (aa) is 8 microns, the theoretical maximum projection error is calculated as follows (Fig. C):

$Instrumental Error (\%)=\left( \frac{c-a}{c} \right) \times100\%$ = $\left( \frac{10-8}{10} \right)=20\%$

We applied this calculation to both manually measured and predicted cilia lengths to estimate the maximum theoretical projection error. The results are as follows:

**Assuming Maximum Possible Cilia Length = 10 microns:**

- Theoretical maximum projection error on manually measured cilia: **15.83%**
- Theoretical maximum projection error on predicted cilia: **32.20%**

***Upper bound vs. actual error***

It is also important to emphasize that the calculated errors represent **upper bounds**—the maximum possible error introduced by the 2D measurement. These are not necessarily the actual errors encountered in every measurement. In many cases, the actual error is much less, depending on the specific geometry and orientation of the cilia relative to the imaging plane. The upper bound calculations provide a worst-case scenario, which is crucial for understanding the potential limitations of the measurement technique but may overestimate the typical error encountered in practice.

***Consistency of errors across brain regions***

It is important to note that these instrumental errors are consistent along the z-axis (tissue thickness) for all brain regions. Since the tissue thickness remains the same across different regions, the projection-related errors will be uniform. Therefore, when analyzing relative cilia lengths between different brain regions, these errors should largely cancel out, allowing for reliable comparative analysis. The focus on relative differences helps mitigate the impact of these errors on the overall conclusions, ensuring that the comparisons remain valid even in the presence of projection-related measurement inaccuracies.

**Supplementary Figures**

**b: Tissue thickness**

**a: Measured 2D length of cilium**

**c: Actual 3D length of cilium**

**Figure A.** Best case scenario resulting in no error

**b: Tissue thickness**

**a: Measured 2D length of cilium**

**c: Actual 3D length of cilium**

**Figure B.** Worst case scenario resulting in the maximum possible error due to projection from 3D to 2D

**a = 8 microns (projected length)**

**c = 10 microns (worst case)**

**Possible cilia orientations**

**Figure C**. Error limitation due to actual maximum length of cilia

**Figure D.** Immunostaining of ADCY3-positive cilia in mouse brain. **(a)** Representative immunostaining of ADCY3 within cilia across various brain regions, with a zoomed-in view of the PFC highlights detailed cilia staining at higher magnification (top right). **(b)** Representative images of co-staining of ADCY3 within cilia with DAPI, showing how cilia extend from the cell body and highlighting the structural distinction between the wider base and narrower tip of each cilium.

(b) Images of co-staining of ADCY3 within cilia with DAPI, illustrating cilia tip/base structures and demonstrating how cilia extend from neuronal cells..


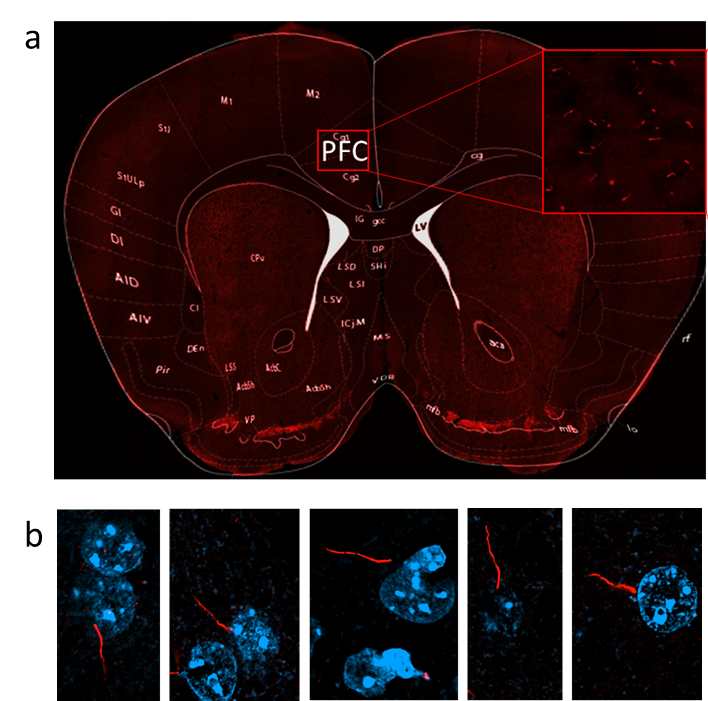

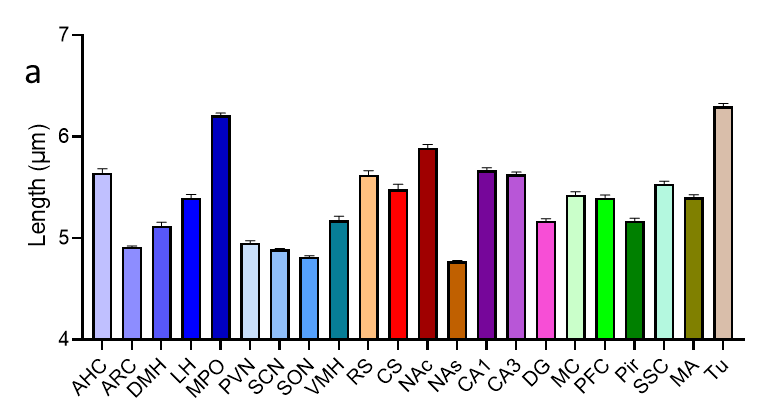

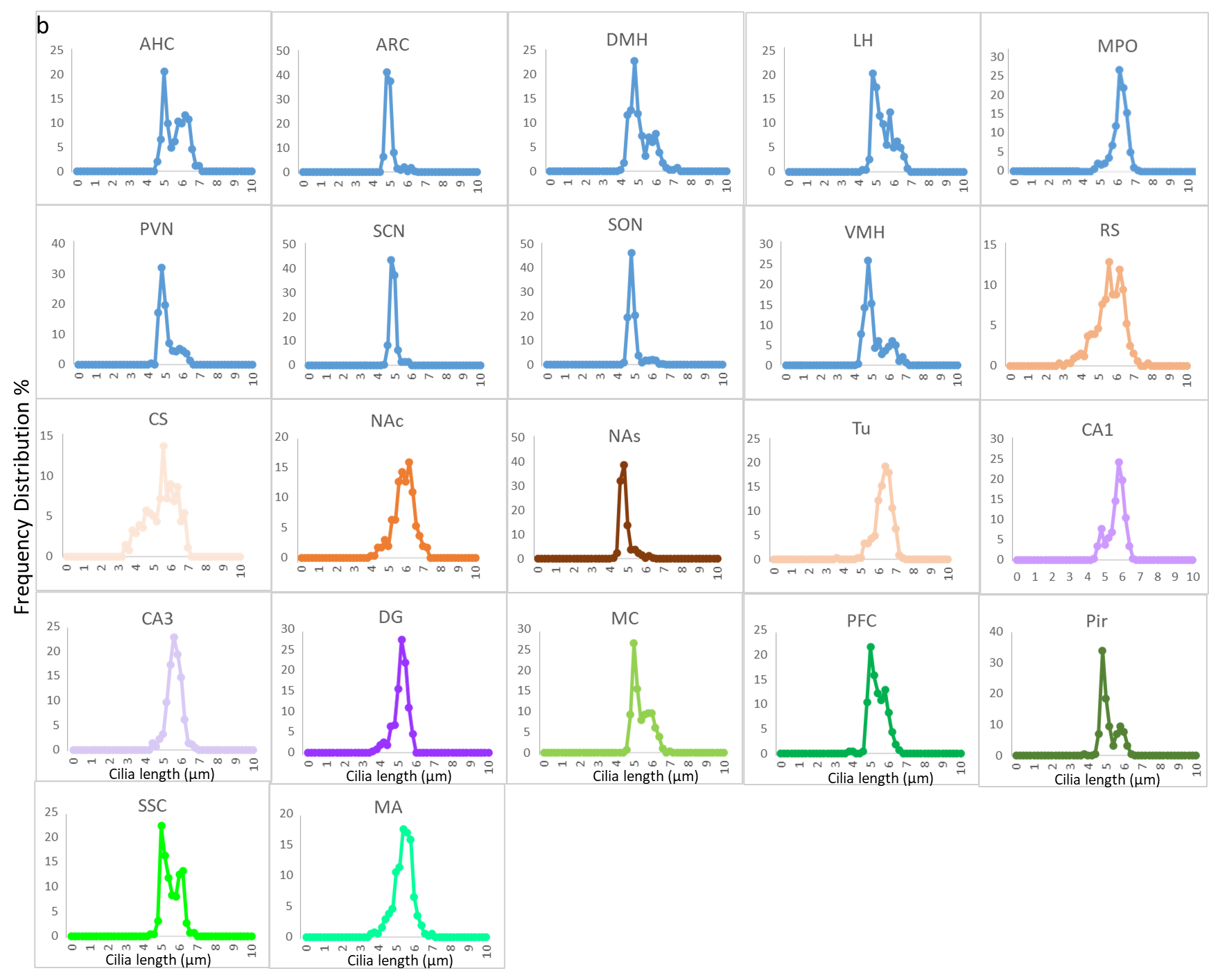


**Figure E (Extended Fig. 2): (a)** Means±SE of cilia length obtained from all measured individual cilia in 22 brain regions; underlying data are available on the Zenodo database (DOI: 10.5281/zenodo.15151271). **(b)** Cilia length frequency of individual brain region section means: Graph showing the distribution of cilia lengths based on the mean lengths calculated from individual brain region section means; underlying data are available in S2 Data.


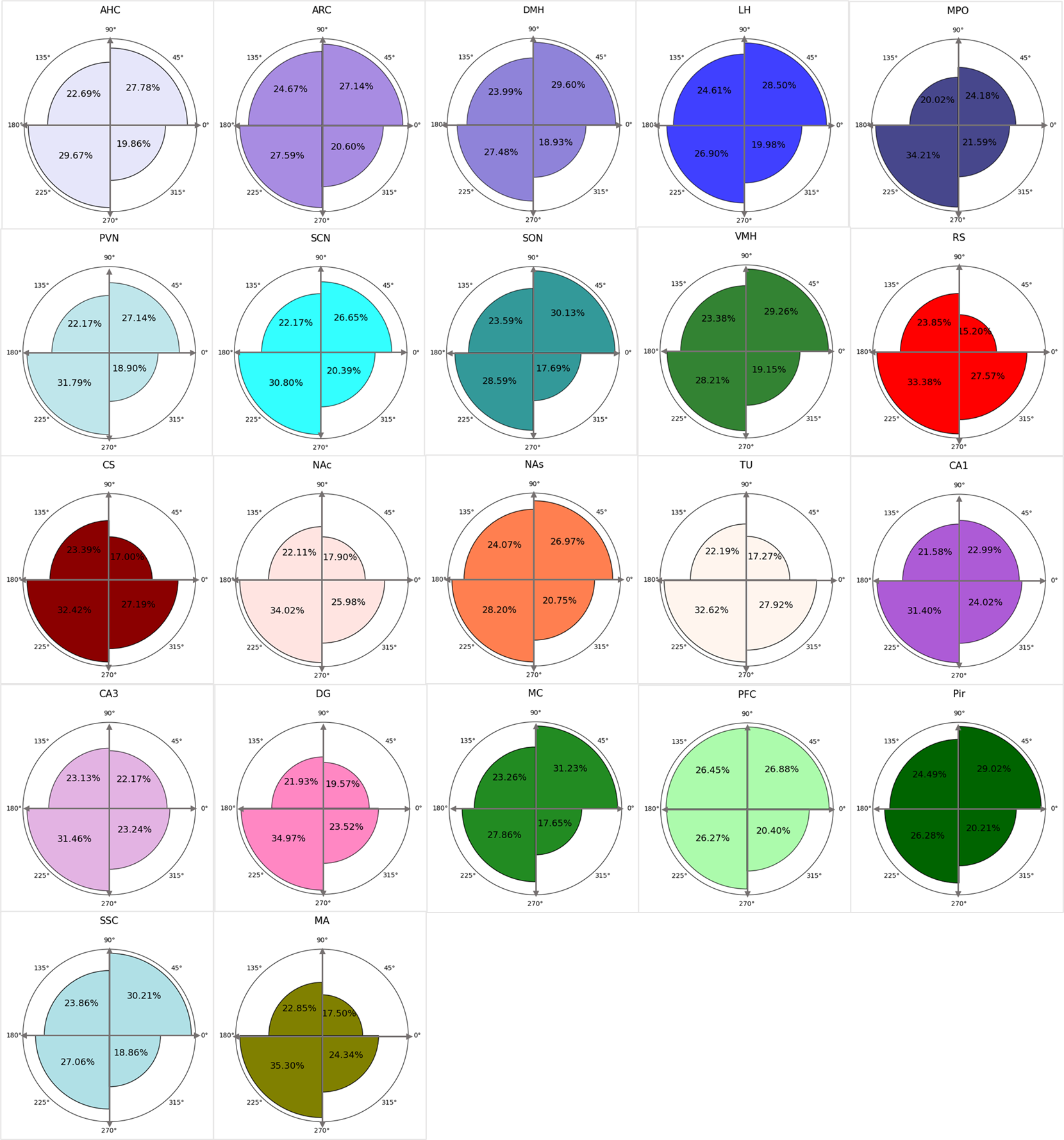


**Figure F. (Extended Fig. 3):** Rose diagram representing the percentage of cilia circular angles in the four polar coordinates of cilia angle distribution. Underlying data are available on the Zenodo database (DOI: 10.5281/zenodo.15151271).


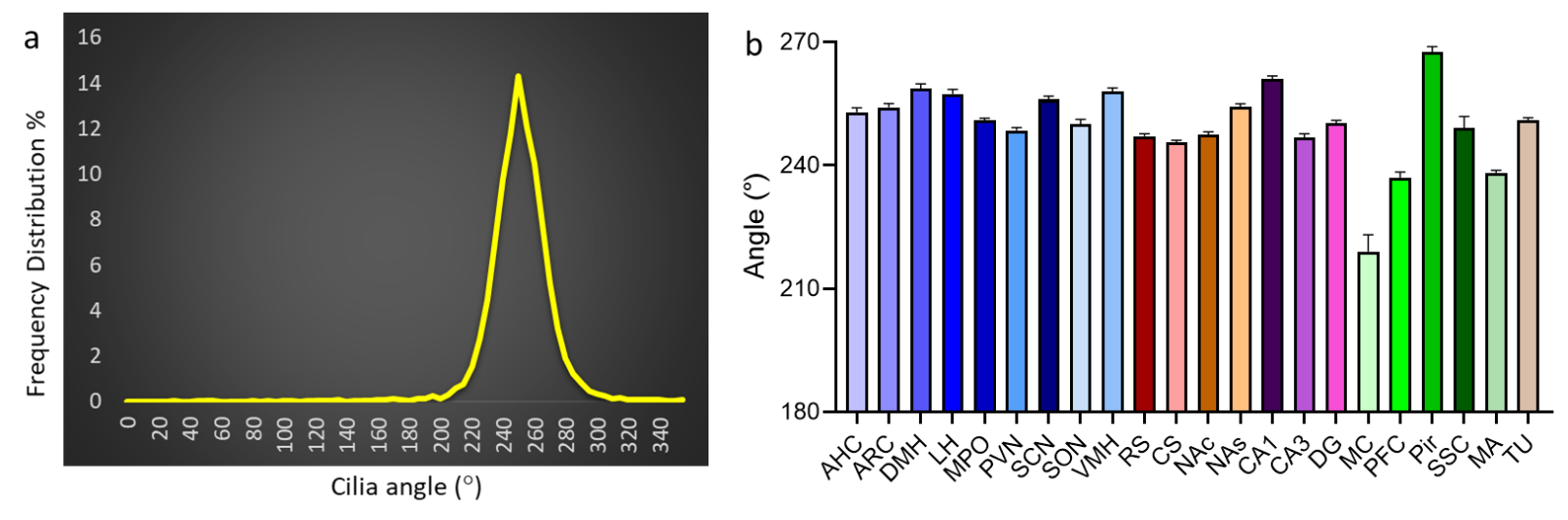


**Figure G. (Extended Fig. 4) (a)** Histogram showing the frequency distribution of cilia circular angles across the entire brain. The analysis is based on section-averaged circular means, where the circular mean of all cilia angles within each section was first calculated and treated as a single value. Underlying data are available in S2 Data. **(b)** Histogram plots of the means±SE of cilia circular angles in the 22 brain regions. Underlying data are available in S2 Data.
